# Nanomaterials in Medicine: Understanding Cellular Uptake, Localization, and Retention for Enhanced Disease Diagnosis and Therapy

**DOI:** 10.14336/AD.2024.0206-1

**Published:** 2024-02-06

**Authors:** Yue Peng, Zhengshuang Yang, Hui Sun, Jinling Li, Xiuwan Lan, Sijia Liu

**Affiliations:** ^1^Collaborative Innovation Centre of Regenerative Medicine and Medical BioResource Development and Application Co-constructed by the Province and Ministry, Guangxi Key Laboratory of Regenerative Medicine & Key Laboratory of Longevity and Aging-related Diseases of Chinese Ministry of Education, Guangxi Medical University, Nanning, Guangxi, China; ^2^Guangxi Colleges and Universities Key Laboratory of Biological Molecular Medicine Research & Guangxi Key Laboratory of Brain Science, Department of Biochemistry and Molecular Biology, School of Basic Medical Sciences, Guangxi Medical University, Nanning, Guangxi, China

**Keywords:** nanomaterials, cellular uptake, subcellular localization, cellular retention, disease applications

## Abstract

Nanomaterials (NMs) have emerged as promising tools for disease diagnosis and therapy due to their unique physicochemical properties. To maximize the effectiveness and design of NMs-based medical applications, it is essential to comprehend the complex mechanisms of cellular uptake, subcellular localization, and cellular retention. This review illuminates the various pathways that NMs take to get from the extracellular environment to certain intracellular compartments by investigating the various mechanisms that underlie their interaction with cells. The cellular uptake of NMs involves complex interactions with cell membranes, encompassing endocytosis, phagocytosis, and other active transport mechanisms. Unique uptake patterns across cell types highlight the necessity for customized NMs designs. After internalization, NMs move through a variety of intracellular routes that affect where they are located subcellularly. Understanding these pathways is pivotal for enhancing the targeted delivery of therapeutic agents and imaging probes. Furthermore, the cellular retention of NMs plays a critical role in sustained therapeutic efficacy and long-term imaging capabilities. Factors influencing cellular retention include nanoparticle size, surface chemistry, and the cellular microenvironment. Strategies for prolonging cellular retention are discussed, including surface modifications and encapsulation techniques. In conclusion, a comprehensive understanding of the mechanisms governing cellular uptake, subcellular localization, and cellular retention of NMs is essential for advancing their application in disease diagnosis and therapy. This review provides insights into the intricate interplay between NMs and biological systems, offering a foundation for the rational design of next-generation nanomedicines.

## 1. Introduction

A new industry that spans the entire modern human production process is the development and research of nanomaterials (NMs), a result of the rapid advancements in nanoscience and nanotechnology. Because of their exceptional physicochemical qualities (distinctive size, shape, and structure, etc.), NMs are extensively employed in the fields of thermal, mechanical, electrical, energy-environmental and life-science [1]. The intensive research and rapid development of NMs has provided easier and more effective solutions to many medical issues. The nanoscale diameters of NMs vary from 1 to around 100 nm [2]. Because of their tiny size, NMs are easily distributed throughout the body and cross biological barriers. Consequently, there is a hope that NMs are promising to reach the site of disease to act and show great potential in both treatment and diagnosis of a wide range of diseases, such as cancer [3], neurodegenerative diseases [4], cardiovascular diseases [5], infectious diseases [6], etc. At the same time, as NMs enter the body, they will inevitably interact with cells. Therefore, the focus of research has moved to biological safety as well as potential threat of the NMs. It is a great challenge to realize the effective uptake of NMs by cells, to reach the specific sites of cells, and to achieve good therapeutic and prognostic effects of the disease [7].

The fundamental building block of life is the cell, and the cell membrane (CM) serves as the channel by which the cell exchanges materials with the outside environment in order to perform a variety of biological functions. CM is a layered structure that contains ion channels, receptors, lipids, enzymes and other functional units. It is a semi-permeable barrier that regulates and chooses which chemicals carry membranes, so isolating cells and their organs from their surroundings [8]. The cell surface receives and reroutes certain signals in order to produce a specific response, such as migration, intravenous action, sputum action, etc. Any cell will respond to changes in the surrounding environment or other cells by triggering the CM to make the corresponding changes in the process of cell activity [9-11]. On the one hand, when NMs enter the cellular environment, the cell influences the fate of NMs, including targeting, internalization, transport, and efficiency of excretion from the body [12-14]. On the other hand, NMs also affect the state of cells [15]. For instance, certain NMs have the ability to either stimulate cell apoptosis or proliferation [16-17]. It is clear that the fields of biomedicine and nanomedicine depend heavily on the interactions between NMs and cells. While a thorough investigation of the interactions' impacts and potential biosafety concerns is necessary, understanding these interactions is crucial for both creating NMs and achieving their medical effects.

The various cellular uptake methods of NMs, such as endocytosis and other modalities, and how they are targeted to particular subcellular structures within the cell will be thoroughly covered in this review. We will also pay particular attention to the variables that affect the intracellular retention of NMs and the ways in which this retention period can be controlled. For the further development and optimization of NMs in the diagnosis and treatment of disease, a thorough understanding of these factors is essential. We may make greater use of the exceptional qualities of NMs and expand their uses in the detection and treatment of disease by thoroughly investigating these mechanisms.

## 2. Fundamentals of Nanomaterials

### 2.1 Unique Properties of Nanomaterials

**Figure 1.**
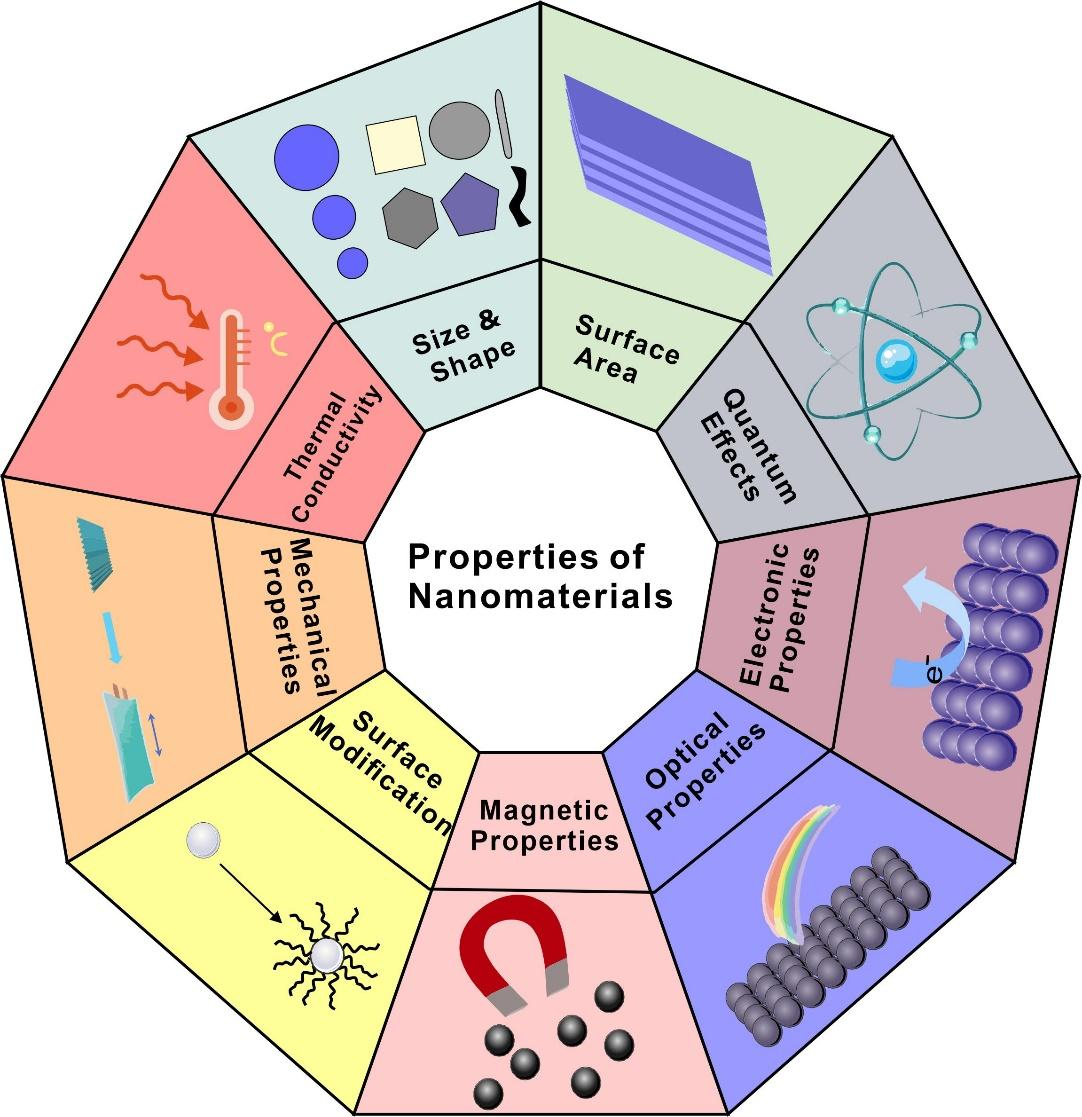
Properties of nanomaterials.

Since "nanotechnology" was first proposed by Richard Feynman, Nobel Prize winner in physics, in a speech in 1959, after decades of development, this emerging interdisciplinary research field has made various revolutionary breakthroughs and is considered to be the innovative technology of the 21st century [18, 19]. NMs, also known as ultraparticulate materials, are interpreted by the International Organization for Standardization (ISO) as "material with any external nanoscale dimension or having the internal nanoscale surface structure" [20]. NMs can be categorized based on their origin (natural or synthetic), chemical composition, material matrix (carbon-based NMs, inorganic matrix NMs, organic matrix NMs, composite matrix NMs), or their dimensionality (0-D, 1-D, 2-D, 3-D) [21]. Nanoparticles (NPs), nanocomposites, nanocolloids, quantum dots, and nanostructure materials (such as nanowires, nanotubes, nanoparts, nanometers, and nanoports) are examples of widespread NMs [22-26]. In order to satisfy the increasing demand for applications, materials science research has recently concentrated on developing novel materials with outstanding qualities (Fig. 1). Because of their numerous advantages, such as their superior physical and chemical properties, flexibility of surface modification, and accessibility of varying size and shape, etc, NMs currently have widespread application in a variety of industries [27] (Fig. 1).

### 2.1.1 Size and Shape

Firstly, the advantage in size makes NMs uniquely attractive in the biomedical field. NMs refer to materials with at least one dimension in the nanoscale range (1-100 nm), constituting a new generation of materials composed of NPs with dimensions between atoms, molecules, and macroscopic systems [28]. The moderate size of NMs, larger than most "small molecule" drugs, allows for prolonged circulation time without vessel blockage, thereby enhancing the bioavailability and pharmacokinetic characteristics of various drugs [29]. Reports have demonstrated the preparation of a series of graphene oxide (GO) samples with different lateral dimensions, validating that smaller NMs are more likely to be absorbed by cells, offering significant advantages when specific molecules face challenges penetrating cells [30]. The size advantage of NMs also includes effective deposition throughout the respiratory tract via diffusion mechanisms when inhaled [31]. Their small size facilitates cellular uptake, allowing them to traverse epithelial and endothelial cells to enter the bloodstream and lymphatic circulation, reaching potential sensitive targets such as bone marrow, lymph nodes, spleen, and the heart [32-34]. NMs have also been observed to translocate into the central nervous system and ganglia via axonal and dendritic pathways [35].

The size of NMs can be adjusted using various synthesis techniques and methods, enabling them to be controllably designed as ideal products with specific functionalities to meet diverse application needs [36]. Currently, materials can be optimized and synthesized using both bottom-up and top-down approaches to achieve the desired size of NMs [37]. For example, Moreno et al. [38] reported a bottom-up synthesis method, producing nanoscale porous graphene with an aperture of approximately 1 nanometer, making it a highly versatile semiconductor. In another study, coconut shell NPs were synthesized through a top-down grinding method, with the size decreasing and surface area increasing as the grinding time increased, resulting in particle agglomeration and the formation of new compounds [39]. Similarly, the morphology of NMs varies with different synthesis methods [40]. Solvent-based, morphology-controllable synthesis methods for C_60_ have been widely researched, adjusting the crystal structure to control the synthesis of different forms of C_60_, including nanowhiskers, nanosheets, nanoballs, microtubes, and spheres [41-43].

### 2.1.2 Specific Surface Area

The miniture size of NMs endows the NMs with a relatively large specific surface area, which provides a huge substrate for specific parts used for active targeting. More chemical molecules can be included with the surface, allowing these small-sized NMs to pass through the CM, especially into the mitochondria [44]. The comparatively large surface area of NMs gives them unique features, such as the ability to act as catalysts in chemical reactions [45]. Particles with a size smaller than 100 nm demonstrated higher biological activity per unit mass when compared to larger particles. The heightened bioactivity can have positive effects, such as antioxidant activity, the capability to carry therapeutic agents, and the ability to penetrate cellular barriers during drug delivery [46, 47]. Conversely, it can also have negative consequences, such as toxicity, the triggering of oxidative stress, or disruption of cellular function [48, 49]. In some cases, bioactivity can exhibit a combination of both positive and negative effects. For instance, the well-distributed amorphous nanosilica with a particle size of 70 nm has a beneficial ability to penetrate the epidermal barrier [50]. Another research found that injecting pregnant mice with silica NPs measuring 70 nm in diameter resulted in pregnancy difficulties. However, bigger sizes of silica NPs (300 and 1000 nm) did not produce similar complications [51].

### 2.1.3 Quantum Effects

The quantum effect is a result of the duality of quantum mechanics and particle waves, and NPs have this effect. When Au reaches the nanoscale, for example, quantum effects will begin to dominate and its color, intermolecular chemistry, and melting temperature will change [52]. At the same time, the quantum size effect is also used to explain the phenomenon of band gap enhancement, and the band gap of the material increases with decreasing size [53]. Quantum effects of NMs are widely used in many fields. In the biomedical field, the Raman scattering signals of molecules can be enhanced due to the localized field strength generated at the surface of the nanostructures, which can be used for biomedical diagnostics. For example, Daniil Nozdriukhin et al. [54] engineered bi-modal contrast agents by integrating carbon nanotubes (CNTs) and gold nanoparticles (AuNPs) around silica microspheres using the Layer-by-Layer self-assembly method. This NMs exhibits extremely high optoacoustic contrast (also called photoacoustics) and Raman scattering and has a good application prospect in diagnosing and locating diseases such as localization photoacoustics tomography.

### 2.1.4 Electronic Properties

Electrons exhibit dual characteristics as both particles and waves, and it is widely accepted that they can flow unrestrictedly within the inner regions of NMs. The characteristics of a substance are determined by the specific movements that its electrons can execute, which are influenced by the available spaces for them to occupy. Consequently, the qualities of a substance are influenced by both its dimensions and configuration [55]. The resistivity of the material is increased by the collision of electrons with the surface of the nano-object, which is caused by quantum phenomena and the significantly high surface-area-to-volume ratio. The resistivity of the NMs is increased by the collision of electrons with its surface, which is caused by quantum phenomena and the material's high surface area to volume ratio. The electronic characteristics of NMs can exhibit substantial disparities compared to macroscopic materials, and in some cases, they can even demonstrate full opposition. This phenomenon plays a crucial role in the development of electronic devices and energy conversion processes [56]. Furthermore, the distinctive electrical characteristic of NMs has found extensive application in various therapeutic domains, including bacterial eradication, wound recovery, and cancer therapy [57]. As an illustration, Cheng et al. [58] created ceria-graphene nanocomposites that contained arginine and could be separated. The overlapping of energy levels between ceria NPs and graphene enables the effective separation of electrons and holes upon exposure to light. This separation leads to the production of reactive oxygen species (ROS), which can effectively eliminate germs. Additionally, it facilitates the migration of fibroblasts to the location of the wound, hence accelerating the process of wound healing.

### 2.1.5 Optical Properties

The optical properties of NMs are based on the electronic structure, and the two are largely interdependent. It is also controlled by the size and shape, firstly owing to the increased energy level spacing due to the additional confinement structure, and secondly due to the surface plasmon resonance (SPR). SPR is generated when the particle size of the metal nanostructure is smaller than the wavelength of the incident radiation [59]. Therefore, these NMs can exhibit special optical phenomena such as absorption and fluorescence [60]. For example, noble metal NMs have size-dependent optical properties, and their unique plasmonic light-absorbing properties have been reported for chemical sensors and biosensors [61].

### 2.1.6 Magnetic Properties

The inhomogeneous distribution of electrons in some NMs leads to their magnetic properties, such as NPs or nanowires, which have special magnetic properties. These NMs are suitable for magnetic storage and biomedical applications such as multiphase and homogeneous catalysis, magnetic fluids, and magnetic resonance imaging (MRI) [62]. The magnetic properties of NPs may also differ from those of related bulk materials. This is mainly due to the smaller size of the particles, the increased surface area, and the increased magnetic coupling with neighboring atoms, which leads to a change in the magnetic properties [63]. Iron oxide is paramagnetic at the nanoscale, but it does not operate in the same way as conventional paramagnetic, so it is called superparamagnetism [64]. The properties of the superparamagnetic NMs are even better. Animals with Walker-256 carcinosarcoma benefited more from the anticancer effects of magnetic nanotherapy because the superparamagnetic NMs responded to resonance conditions [65].

### 2.1.7 Surface Modification

The surface of the NMs can be customized to achieve a specific function. For example, the surface modification of NPs with specific antibodies or peptides can achieve the effect of tissue targeting, thereby reducing the targeting efficiency of NMs and enhancing the toxic effect in vivo [66]. Surface customization modifications in drug delivery such as coating with hydrophilic polymers confer stealth properties to avoid elimination by the reticuloendothelial system, thereby prolonging circulation time and increasing drug concentration at the site of action [67]. At the same time, the solubility of NMs is usually achieved by surface modification. This soluble substance, which can be dispersed in different solvents, helps to solve the problems of solubility and stability of insoluble drugs [68].

### 2.1.8 Mechanical Properties

The outer surface of nanostructures has few or no defects, and materials with fewer defects will exhibit more advanced mechanical properties than bulk materials, such as mechanical hardness, elastic modulus, scratch resistance, fracture toughness, etc. [69]. One representative material is bacterial nanocellulose (BNC), which is produced by mechanically or chemically breaking down cellulose fibers into individual nanoscale components. The unique physical properties such as excellent mechanical strength and light weight have led to a wide range of applications of nanocellulose in fields such as biomedicine and materials science [70]. The BNC-hydrogel NMs developed by Nimeskern et al. [71] has a mechanical modulus similar to that of human cartilage. They showed that these composites can be transformed into complex, specific shapes. In addition, Rueda et al. [72] designed polyurethane-cellulose nanocrystal nanocomposites with high mechanical strength and good ductility through in-situ polymerization to support the proliferation of fibroblasts.

### 2.1.9 Thermal Conductivity

Size also affects the thermal conductivity of NMs. As particle size decreases, melting entropy and enthalpy fall as well, while specific heat increases, according to studies [73]. One potential use of NMs' thermal conductivity is in nanofluids, which can enhance heat transmission [74]. It is anticipated that fluids with suspended nanoparticle solid particles will exhibit thermal conductivity that is substantially higher than that of typical thermal conductivity fluids. Nanofluids made of CuO or Al_2_O_3_ NPs in ethylene or water have high thermal conductivity, as demonstrated by Cao et al. [75]. The growth of real-time, directly intrusive medical diagnostic systems and the development of revolutionary diagnostic and therapeutic techniques for cancer and other diseases are both aided by devices based on nanofluids [76].

The characteristics of these NMs give them a wide range of potential applications in various fields, such as electronics, materials science, and biomedicine. Different types of NMs may exhibit different characteristics, so their unique properties need to be fully considered when selecting and designing NMs.

### 2.2 Applications of Nanomaterials in Various Industries

As mentioned earlier, due to their unique physicochemical properties such as nanoscale size, specific surface area, photoelectric activity, magnetism, mechanical strength, and thermal conductivity, NMs find applications in various fields. In the electronics sector, NMs hold tremendous potential for developing smaller, faster, and more efficient electronic devices. Various functional inks containing NMs, such as metals, organic electronic molecules, CNTs, and ceramic NPs, are expected to rapidly become the large-scale production processes for new electronic devices [77]. Simultaneously, the development, testing, and application of NMs create new directions for the advancement of nanophotonic materials, molecular devices, and quantum structure novel devices, leading to increased efficiency in electronic systems [78]. In environmental science, NMs play a significant role in water pollution control due to their effective adsorption capabilities. Studies indicate that nanomembranes and filters based on CNTs, nano-porous ceramics, magnetic NPs, and other NMs can effectively remediate water pollution through nanofiltration [79]. Biochemical nanosensors can detect tiny organic pollutants and toxins, while carbon nanotube nanocomposites can serve as efficient gas adsorbents for environmental remediation [80]. Moreover, solar energy storage and conversion systems based on NMs address the issues of low efficiency and high costs associated with traditional solar cells [81, 82]. This has made a significant contribution to the development of green energy technologies and environmental protection.

It is noteworthy that NMs are of interest to all branches of medicine for their ability to deliver drugs within the optimal dosage range, as they can effectively deliver the drug to the target area, thereby increasing therapeutic effectiveness while reducing side effects and improving patient dependence [83, 84]. A number of lipid NMs have also been used as drug carriers and release RNA in the treatment of cancer [85, 86].Ultrasonically magnetic iron oxide NMs can be coupled with multiple particles, such as antibodies, can be used as drug carriers for cancer treatment, or as MRI imaging agents for tumor detection [87, 88].This involves the application of NMs in medical imaging, allowing us to obtain accurate anatomical information in different ways.One study showed that magnesium oxide and dianol polymers and magnetoliprose NPs can act as positive MRI imaging agents and cell tracking markers in Hepa 1-6 cell family [89].And the gold NMs, due to its optical properties enhanced by SPR, allows it to be used as a photothermal agent, using high temperatures to kill tumor cells [90].The application of nanobiotechnology has also opened up a new pathway to the treatment of viruses, and so far gold, silver, sulphide, mercury oxide, magnesium, graphene and certain bio-polymer compounds are the most suitable NMs for diagnosing and effectively combating the new coronavirus [91].

**Figure 2.**
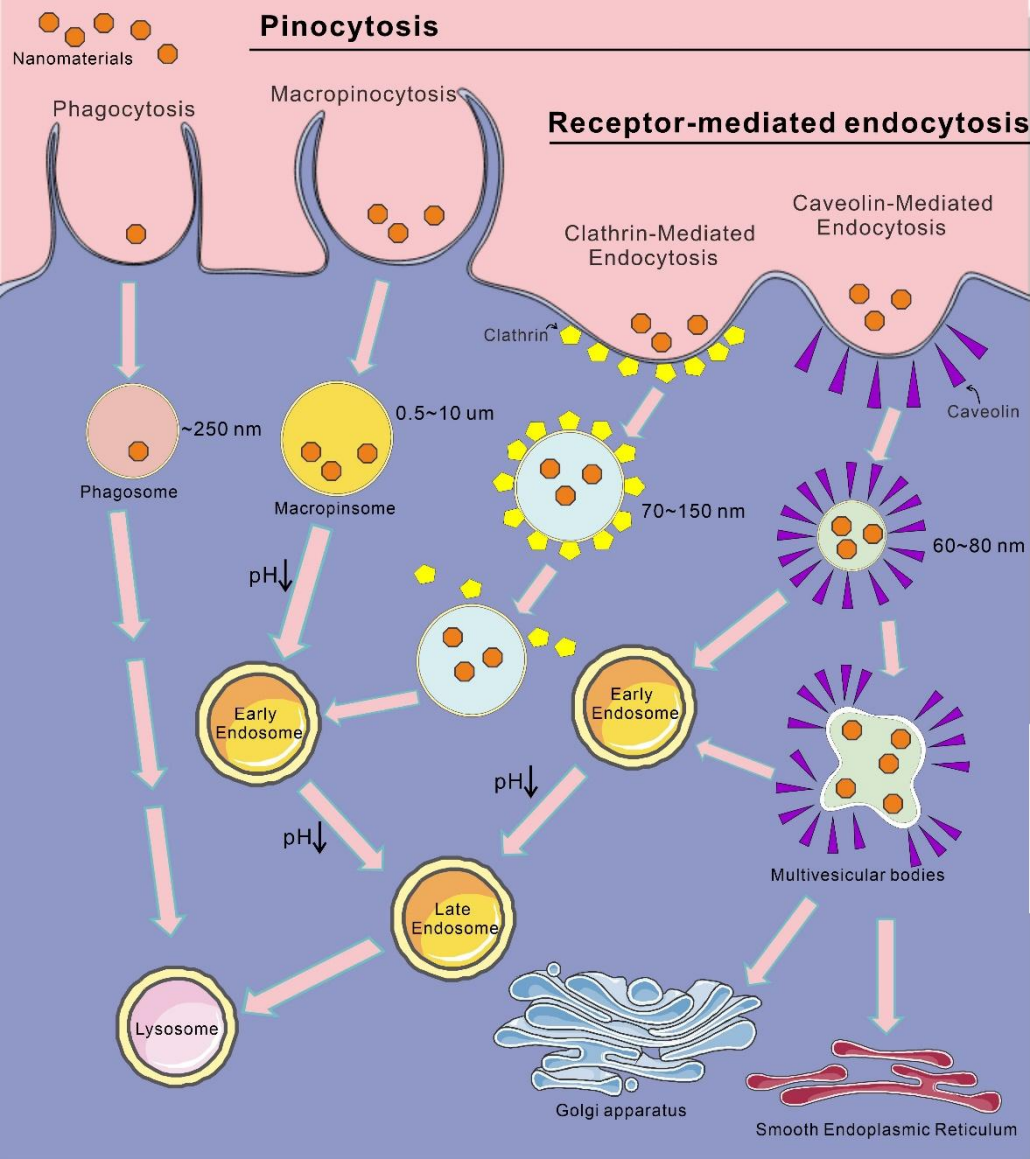
Four major endocytic mechanisms of nanomaterials.

While the rapid advancement of nanotechnology and the widespread use of NMs in medicine offer expanded options and possibilities for disease diagnosis and treatment, it is imperative to thoroughly characterize the potential impact of any NMs on human health and the environment. The investigation into cellular absorption mechanisms is pertinent across various NMs and applications, spanning industries from electronics and environmental science to biomedicine. Within the human body, NMs that exhibit high biocompatibility can interact seamlessly without inducing adverse events, such as toxicity, immune reactions, thrombosis, or cancer. However, when these biological responses are either unresponsive or excessive, NMs may lead to toxicity. Therefore, there is a pressing need for a comprehensive understanding of nanotoxicology, delving into the mechanisms of NMs absorption by cells, their localization within cells, and the duration of their presence. This knowledge is essential to assess potential toxicity and biocompatibility, unlocking the full potential of NMs in diverse biomedical applications. By acquiring such insights, researchers can design safer NMs, predict their biological effects, and steer future research directions in nanotoxicology and nanomedicine. Ultimately, this information aids in advancing the responsible and effective utilization of NMs in medical applications.

## 3. Cellular Uptake of Nanomaterial

When NMs reach the outer membrane of a cell, they can interact with components of the plasma membrane or extracellular matrix (ECM), facilitating their entry into the cell. This process is primarily achieved through endocytosis, where NMs are enveloped by the membrane, forming endocytic vesicles that are subsequently transported to specialized intracellular sorting and transport compartments. This article explores four major endocytic mechanisms (Fig. 2): phagocytosis, micropinocytosis,clathrin-mediated endocytosis (CME), and caveolin-mediated endocytosis (CVME). Phagocytosis predominantly occurs in specific phagocytic cells, while macropinocytosis is more ubiquitous, found in various cell types. Additionally, at the end of this section, the impact of NMs with different properties on cellular uptake will be briefly discussed (Fig. 2).

## 3.1 Mechanisms of Cellular uptake

### 3.1.1 Phagocytosis

Phagocytosis is a cellular mechanism in which a cell surrounds and engulfs large particles by forming a membrane around them, leading to their active internalization. One of the key features of phagocytosis is the presence of internalized vesicles, often referred to as phagosomes, with a typical size of approximately 250 nm [92]. Monocytes, macrophages, neutrophils, dendritic cells, and other cells frequently utilize this mechanism to ingest NMs and microorganisms [93, 94]. The process of phagocytosis is widely used by mammals to absorb microbes, aged cells, and cell debris [95]. The key receptors involved in phagocytosis include the IgG Fc receptor family (FcγRI, FcγRIIA, and FcγRIIIA), complement receptors (CR1, CR3, and CR4), and the α5β1 integrin [96]. Subsequently, the phagosomes containing substances fuses with the lysosome. However, only a limited number of cells utilize phagocytosis as a mechanism for the uptake of NMs.

### 3.1.2 Pinocytosis

Phagocytosis refers to the engulfment of liquid containing solutes and particles within vesicles, with vesicle sizes smaller than those generated during the process of phagocytosis. This mechanism of endocytosis can be categorized into receptor-mediated endocytosis (RME) and macropinocytosis [97]. RME is the most common pathway for NMs entry into the cell. RME initiates with the binding of ligands attached to NMs to specific receptors on the CM, triggering a conformational change leading to membrane invagination and the formation of early endosomes. RME encompasses different types, primarily classified as CME, CVME, and clathrin/caveolin-independent endocytosis.

CME is the most extensively studied pathway for transporting substances into eukaryotic cells [98]. CME is a complex process involving intercellular signal transduction, membrane recycling, and nutrient absorption. The formation of vesicles involves a wide array of proteins [99-103], interactions between stimulants and their receptors, and the encapsulation of vesicles by clathrin to form clathrin-coated vesicles. The size of clathrin-coated vesicles depends on the cell type and typically ranges from 70 to 150 nm [104]. The release of vesicles from the plasma membrane is regulated by GTPase and dynamin [105-107]. Upon vesicle release, clathrin is degraded by auxilin and heat shock cognate 70-dependent proteins [104]. Kannan et al. [108] found that polystyrene NPs typically enter cells through CME [109]. Most lipid-based NMs enter cells via CME, such as in UROtsa human urothelium bladder cells, A431 human epidermoid cancer cells [110]. Following internalization via CME, the clathrin coating is dissociated, with a portion directed to early endosomes and another portion recycled to the plasma membrane surface. Vesicles can also target later-stage endosomes, then proceed to lysosomes and multivesicular bodies (MVBs), among other compartments. The cellular uptake of most receptor-mediated NMs occurs through CME.

CVME stands as the second most extensively investigated endocytic pathway and has been demonstrated to play a crucial role in intracellular transport and mechanosensing across cells. Triggered by ligand-receptor binding, CVME results in the formation of flask-shaped invaginations, known as caveolae, with a diameter of 60-80 nm in the plasma membrane [111]. Various proteins participate in this endocytic pathway, such as caveolin-1, which associates with cholesterol in lipid rafts; notably, unlike CME, caveolin-1 remains associated with vesicles after internalization [112]. The formation of vesicles is also regulated by kinases and phosphatases, including Src tyrosine kinase and serine/threonine protein phosphatases PP1 and PP2A [113]. Similar to CME, dynamin is required to facilitate vesicle detachment from the plasma membrane, forming caveolin vesicles that subsequently fuse with other caveolin vesicles to generate vesicles with a multivesicular structure, further fusing with early endosomes [114]. Depending on cell type, vesicles can move to the smooth endoplasmic reticulum (ER) or the Golgi transport network [115]. When Liu et al. incubated iron oxide nanoparticles (IONPs) synthesized by them with mouse macrophage (RAW 264.7 cells) and human ovarian cancer cells (SKOV-3 cells), they observed that the NPs primarily entered cells through the CVME mechanism [116].

Macropinocytosis is a dynamic cellular internalization process in which cells actively engulf substantial volumes of extracellular fluid, forming large vesicles referred to as macropinosomes with diameters ranging from 0.5 to 10 μm [117]. This cellular process is a typical mechanism for macrophages to ingest apoptotic cell fragments, cells, and bacteria [118]. Unlike phagocytosis and RME, macropinocytosis is not directly regulated by receptors or other molecules. Studies indicate that activation of tyrosine kinase receptors leads to the formation of macropinosomes during macropinocytosis [119]. Following the internalization of NMs through macropinocytosis, the pH within macropinosomes gradually decreases, and markers for late endosomes begin to appear. Subsequently, acidified macropinosomes can fuse with late endosomes, associate with lysosomes, or recycle their contents back to the CM [120].

Furthermore, there are pathways notably Clathrin-independent endocytosis (CIE) that are not reliant on clathrin or caveolin, in addition to the processes outlined before. There is evidence that CIE regulates processes such as cell polarization, intercellular signal transduction, cell growth, and plasma membrane repair [121]. In endocytosis, several proteins are involved in the CIE process, such as Arf-6, Rho-A (or IL2Rb-dependent pathway), flotillin, and CDC42 (CLIC/GEEC) [122, 123]. Proteins important in amino acid absorption and ECM interactions, including β-integrin and glucose transporter GLUT1, are internalized via Arf-6-dependent CIE [124, 125]. In order to produce vesicles, endocytosis regulated by RhoA and CDC42 depends on the creation of raft proteins. RhoA is a route that relies on dynamin and is involved in the process of immune cell and fibroblast protein internalization, including the interleukin-2 receptor (IL-2R-β) and others [126]. Research indicates that CDC42, which is a dynamin-independent pathway, is involved in the absorption of CtxB and VacA, on the other hand [127, 128]. Unfortunately, this study does not delve further into these routes because there is a lack of literature on their significance to NMs uptake.

### 3.2 Nanomaterial Characteristics Impact Cellular Uptake

The characteristics of NMs play a crucial role in influencing cellular uptake, including size, shape, charge status, and surface modifications (Table 1). These aspects are intertwined, collectively shaping the dynamic and intricate interactions between NMs and cells.

Size serves as the foundation for the interactions between NMs and cells. Smaller NMs typically find easier passage through micropores on the CM or internalize into cells. Reports suggest that NMs below 150 nm are predominantly internalized through CME or CVME, while NMs ranging from 250 nm to 3 μm are absorbed through macropinocytosis and phagocytosis [129]. Cui et al. investigated the cellular uptake of single-walled carbon nanotubes (SWCNTs) of varying lengths [130]. The experiments revealed that the predominant cellular uptake mechanism for SWCNTs is macropinocytosis, followed by CVME and CME. In contrast, very short SWCNTs (50 nm or smaller) have the capability to directly insert into the CM and diffuse into cells [131]. The cellular uptake of NMs based on alginate also depends on size. Studies demonstrate that alginate oil ester NPs of sizes 50 nm and 120 nm enter cells through CME, while 420 nm NPs enter cells through CVME, and 730 nm particles are absorbed by macrophages in human colorectal cancer cells (Caco-2 cells) [132].

Simultaneously, the shape of NMs is also a critical factor. Different shapes can influence their contact and interaction with the CM, thereby affecting uptake efficiency. Mitragotri and colleagues discovered that the geometric shape of NMs can impact cellular internalization [133, 134]. NMs of different shapes create distinct angles between the membrane and particles at cell adhesion points. This contact angle significantly influences the ability of macrophages to internalize particles through actin-driven membrane movements. Mitragotri et al. studied six different shapes of NPs: spheres (radius 1.0-12.5 μm), prolate ellipsoids (major axis 4 μm, aspect ratio 4), oblate ellipsoids (major axis 2-6 μm, aspect ratio 1.3-3), elliptical disks (major axis 3-14 μm, aspect ratio 2-4, thickness 400-1000 nm), rectangular disks (major axis 4-8 μm, aspect ratio 1.5-4.5), and UFOs (spherical radius 1.5 μm, annular radius 4 μm). The experiments demonstrated that NMs with high aspect ratios are less prone to cellular engulfment, and elongated, pointed particles are internalized faster than flat particles [135]. Conversely, pancake-shaped NMs, due to their flattened morphology, attach to the CM like a "backpack", blocking the entrance to the cell [136, 137]. Polymeric nanospheres with an elliptical disc shape exhibit smaller contact angles, allowing them to vertically attach along the CM axis and become engulfed by the membrane [134].

**Table 1:** Impact of nanomaterial characteristics on cellular uptake.

Nanomaterials	Size	Shape	Surface charge	Surface modifications	Cellular uptake mechanism	Ref.
**Long single-walled carbon nanotubes (L-SWCNTs)**	630 ± 171 nm	——	——	——	Macropinocytosis> caveolae-mediated endocytosis (CVME)> clathrin-dependent endocytosis (CME)	[130]
**Medium single-walled carbon nanotubes (M-SWCNTs)**	390 ± 50 nm					
**Short single-walled carbon nanotubes (S-MWCNTs)**	195 ± 63 nm					
Single-walled carbon nanotubes (SWNTs)	100-200 nm	——	——	——	Clathrin-coated pits	[131]
	**50-100 nm**				Clathrin-coated vesicles as well as the caveolae pathway	
	**Within 50 nm**				Insertion and diffusion	
Oleoyl alginate ester (OAE) nanoparticles	50 nm/120 nm	——	——	——	Clathrin-mediated endocytosis (CME)	[132]
	**420 nm**				Caveolae-mediated endocytosis (CVME)	
	**730 nm**				Macropinocytosis	
**Polystyrene (PS) particles**	——	Worm-like	——	——	Endocytosis or pinocytosis pathway (non-phagocytic cells)	[133]
Polystyrene (PS) particles	Radius 1.0-12.5 μm	Spheres	——	——	Macropinocytosis	[134]
	**Major axis 4 μm, aspect ratio 4**	Oblate ellipsoids				
	**Major axis 2-6 μm, aspect ratio 1.3-3**	Prolate ellipsoids				
	**Major axis 3-14 μm, aspect ratio 2-4, thickness 400-1000 nm**	Elliptical disks (EDs)				
	**Major axis 4-8 μm, aspect ratio 1.5-4.5**	Rectangular disks				
	**Sphere radius 1.5 μm, ring radius 4 μm**	UFOs				
Colloidal gold nanoparticles	74 × 14 nm	Rod-shaped	——	——	Receptor-mediated endocytosis	[135]
	**74 or 14 nm**	Spherical				
Water-soluble manganese ferrite nanoparticles (MFNPs)	138.9 ± 69.4 nm	——	Non-ionic (-11.9 ± 0.9 mV)	——	Macropinocytosis	[138]
	**117.0 ± 53.6 nm**		Cationic (37.2 ± 1.0 mV)			
	**182.1 ± 88.3 nm**		Anionic (-27.0 ± 4.2 mV)			
Iron oxide nanoparticles	136 nm	——	-59 mV	Silica-coated superparamagnetic iron oxide nanoparticles (SPIONs)	Caveolae-mediated endocytosis (CVME)	[141]
	**133 nm**		-14 mV	PEGylated SPIONs	Caveolae-mediated endocytosis (CVME) and CDC42-mediated endocytosis	
	**17 nm**		50 ± 5 mV	Silica-coated iron oxide nanoparticles (SCIONs)	Caveolae-mediated endocytosis (CVME)	
Poly(ethylenimine)s (PEIs)	——	——	——	Branched PEI	Caveolae-mediated endocytosis (CVME)	[142]
				**Linear PEI**	Clathrin-mediated endocytosis (CME)	
**C_60_ fullerenes**	1 nm	Icosahedral	- 44.3 mV	——	Through the passive entry and integration of C_60_ particles into biological membranes	[143]
**C_60_ fullerenes**	161.8 nm	——	——	——	Clathrin-mediated endocytosis (CME)	[144]

The charge of NMs also influences cellular uptake, with positively charged NMs typically having a greater propensity to interact with negatively charged structures on the CM, thereby promoting internalization [138, 139]. For example, research suggests that positively charged zinc oxide NPs exhibit more cellular internalization compared to negatively charged polyacrylic acid-coated zinc oxide NPs [140].

Different surface modifications of NMs also affect the process of cellular internalization. For instance, silica-coated IONPs enter HeLa cells through CVME, while polyethylene glycol (PEG)-modified IONPs enter HeLa cells through CVME and CDC42-mediated endocytosis [141]. Data suggests that NPs with polyethyleneimine (PEI) branched modifications may enter cells through the CVME mechanism, while linear PEI is primarily taken up by cells through the CME pathway [142]. Unmodified C_60_ fullerene (approximately 1 nm in size) can enter RAW 264.7 cells through passive diffusion [143]. However, once modified, the cellular uptake mechanism changes. In human embryonic kidney epithelial cells (HEK293), C_60_ fullerene coupled with phenylalanine/polylysine derivatives enters cells through flotillin, while in 3T3 L1 mouse fibroblasts and RH-35 rat hepatoma cells, C_60_ fullerene is mainly taken up through CME [144].

## 4. Subcellular Localization of Nanomaterials

The precise targeting of NMs to various cellular organelles is a critical focus in biomedical research and therapy. In addition to using signal peptides as targeting molecules, there are several other methods to achieve the targeted entry of NMs into different organelles, such as antibody targeting, nucleic acid guidance, functionalized NMs, and more. This section will summarize the current methods for NMs to target various cellular organelles (Table 2).

### 4.1 Endosomes/Lysosomes

The endosome is a membrane-bound organelle within eukaryotic cells, characterized as a vesicular structure. Typically, the cellular processing of ingested NMs mirrors the handling of internalized biomacromolecules such as proteins, carbohydrates, nucleic acids, and lipids. Upon cellular uptake, NPs are often enclosed within vesicles known as endosomes. Endosomes can undergo various processes, including maturation, characterized by the transition from early to late endosomes, with these distinct stages often accompanied by a gradual decrease in endosomal pH. Ultimately, acidified endosomes may fuse with lysosomes, forming the endolysosomal system, where enclosed NMs are enzymatically digested and degraded [145]. Some endosomes undergo recycling, returning to the plasma membrane through the perinuclear region or directly recycling to the CM, leading to the excretion of encapsulated NMs. Endosomes only break down on occasion, allowing the encapsulated NMs to escape into the cytoplasm [12].

The principal digesting region of the cell is located within lysosomes, which are cellular organelles defined by their acidic interior. Along with certain membrane proteins, they include a wide variety of acid hydrolytic enzymes, including phosphatases, nucleases, glycosidases, proteases, peptidases, sulfatases, and lipases. Endocytosis, exocytosis, autophagy, repair of the plasma membrane, maintenance of cholesterol levels, and phagocytosis are all basic cellular processes in which lysosomes play a role [146, 147]. Lysosomes also serve as the ultimate destination for the degradation of most cellular intake products and large molecules [148].

**Table 2:** The methods for nanomaterials to target cellular organelles.

Cellular organelles	Nanomaterials	Targeting methods	Ref.
Endosomes/Lysosomes	Gold nanoparticles (AuNPs)	Protein corona-mediated nanoparticle binding, internalization, and intracellular transportation	[149]
	**Asymmetric poly (ethyleneglycol)-b-poly (2,4,6-trimethoxybenzylidene-1,1,1-tris (hydroxymethyl)ethane methacrylate)-b-poly (-acrylic acid) (PEG-PTTMA-PAA) triblock copolymers load hydrophilic doxorubicin hydrochloride (DOX·HCl)**	The pH-sensitive degradable chimeric polymer enters the lysosome, and the acetal is hydrolyzed, resulting in the disintegration of the micelle and drug release	[150]
	**Selenium-rubyrin (NMe_2_Se_4_N_2_)-loaded nanoparticle functionalized with folate (FA) (FA-NMe_2_Se4N_2_ NPs)**	Receptor-mediated cell-specific and acidic pH-activated targeting	[151]
	**Squalenyl-Penicillin G Conjugates (pH-sensitive and pH-insensitive) (SqPNG NPs)**	Benzyl-penicillin (PNG) linked to squalene (Sq) through pH-sensitive or pH-insensitive chemical bonds, can be cell internalized through clathrin-dependent and -independent endocytic pathways.	[152]
	**PLLeu-PLL (DMA)-Tat (SA) (PPDTS) micelles**	A pH-sensitive and charge-reversible peptide micelle that gradually responds to the mildly acidic pH of the extracellular tumor microenvironment and endosomes / lysosomes	[153]
Cytoplasm	Multi-component siRNA nanocomplex	Endosomal pathway based on endocytosis	[154]
	**Gold nanoparticles (AuNPs)**	Direct translocation pathways	[155]
	**Zwitterionic thiol ligand D-penicillamine coated quantum dots (DPA-QDs)**	Direct translocation pathways	[156]
	**Gold nanoparticles (AuNPs)**	Direct translocation pathways	[157]
	**Porous silicon nanoparticles (pSiNPs)**	Lipid fusion	[161]
	**Amphiphilic gold nanoparticles (amph-NPs)**	Lipid fusion	[162]
	**Mesoporous silica-coated hollow manganese oxide (HMnO@mSiO_2_) nanoparticles**	Electroporation	[164]
	**Lipid nanoparticles (LNP) mediated siRNA delivery (siRNA-LNP)**	Electroporation	[165]
	**CdSe/ZnS carboxyl-coated quantum dots**	Microinjection	[166]
Nucleus	Ultrasmall gold nanoparticles (AuNPs)	Passive diffusion	[168]
	**Cisplatin**	Passive diffusion	[171]
	**Poly (oligoethylene glycol methacrylate)-block-poly(styrene-co-vinylbenzaldehyde) P(OEGMA)-b-P(ST-co-PVBA) block copolymer nanoparticles**	Passive diffusion.	[172]
	**TAT peptide-conjugated mesoporous silica nanoparticles (MSNs-TAT)**	Active transport	[176]
	**Nuclear localization sequence (NLS) modified Chitosan nanoparticles (NPs)**	Active transport	[181]
	**Nanoconstructs composed of nucleolin-specific aptamers and gold nanostars (Apt-AuNS)**	Active transport	[183]
Mitochondria	Doxorubicin (Dox) conjugated mitochondria-penetrating peptides (MPPs) (mtDox)	Mitochondrial targeting signal peptides	[187]
	**Mitochondria-targeted chlorambucil (mt-Cbl)**	Mitochondrial targeting signal peptides	[188]
	**Mitochondrial targeting signal peptide (MTS) conjugated DOPE lipid) (MTS-DOPE)**	Mitochondrial targeting signal peptides	[194]
	**N-(2-hydroxypropyl) methacrylamide (HPMA) copolymers-doxorubicin (DOX)-cell penetrating peptide R8-mitochondrial targeting sequence ALD5^MTS^(P-D-R8MTS)**	The combination of mitochondrial-targeting sequences with CPPs effectively targets the mitochondria.	[196]
	**Triphenoylphosphonium (TPP)-functionalized mesoporous silica nanoparticles (MSNPs) (MSNP-PPh_3_)**	Lipophilicity and delocalized cationic properties	[197]
	**Triphenylphosphonium (TPP)-Membrane lytic peptides (MLP)-arginine-glycine-aspartic acid (RGD)(TPP-Lytic peptide-RGD)**	Lipophilicity and delocalized cationic properties	[198]
	**Triphenyl phosphonium (Mito-FF) fibrous nanostructure**	Lipophilicity and delocalized cationic properties	[199]
	**Bifunctional mitochondria-targeted anticancer agent (FPB)**	Lipophilicity and delocalized cationic properties	[201]
	**Cationic plastoquinone derivatives (SkQs)**	Lipophilicity and delocalized cationic properties	[205]
	**Single-walled carbon nanotubes (SWNTs) functionalized with phospholipid-polyethylene glycol (PL-PEG) (SWNT-PL-PEG)**	Exploiting the mitochondrial membrane potential	[209]
	**Single-walled carbon nanotubes (SWNTs)**	Exploiting the mitochondrial membrane potential	[210]
	**Single-walled carbon nanotubes (SWNTs)**	Exploiting the mitochondrial membrane potential	[211]
	**Single-walled carbon nanotubes (SWCNTs)**	SWCNTs deoxidized cytochrome c in mitochondria in a pH-dependent manner.	[212]
	**Fluorescent nanocrystal quantum dots (QDs)**	Mitochondrial targeting signal peptides	[213]
	**Iron Oxide Nanoworms Coated with CGKRKD[KLAKLAK]_2_ (CGKRK_D_[KLAKLAK]_2_-NWs)**	Mitochondrial targeting signal peptides	[214]
	**Liposome (MITO-Porter system)**	Membrane fusion mechanism	[215]
Endoplasmic Reticulum	Phospholipid bilayer covered saquinavir (SQV) pure drug NP (Lipo@nanodrug)	Lipophilicity	[217]
	**Metal (M)-, N-, and O-doped carbon-dominated nanoparticles (MNOCNPs, M-=-Ni, Pd, or Cu)**	Poly (ethylene glycol) (PEG) modification	[218]
	**Amphiphilic quinoxalinone derivative-peptide nanodot (Q1-PEP)**	A highly stable aggregation-induced emission (AIE) fluorescent nanodot assembled by an amphiphilic quinoxalinone derivative-peptide conjugate exhibits large Stokes shift and an endoplasmic reticulum (ER)-targeting capacity	[219]
	**Gold nanoparticles (AuNP)- KDEL (Lys-Asp-Glu-Leu) peptide-siRNA (Au-KDEL-siRNA)**	ER-targeting peptides	[223]
	**Anti-KDEL functionalized polymeric nanoparticles (NPs) loaded with paclitaxel (Tx) (NPs-Tx-KDEL)**	ER-targeting peptides	[224]
	**PLGA nanoparticless loaded with peptide**	ER-targeting peptides	[227]
	**Pardaxin-Indocyanine green-loaded gold nanospheres (FAL-ICG-HAuNS nanosystem)**	ER-targeting peptides	[228]
Golgi apparatus	Retinoic acid (RA)-conjugated chondroitin sulfate (CS) loading with paclitaxel (PTX) (PTX-CS-RA)	High affinity compound surface modification	[230]
	**Doxorubicin (DOX)+retinoic acid (RA)-chondroitin sulfate nanomicelles (CSmicelles) codelivery system**	High affinity compound surface modification	[231]
	**All-trans retinoic acid loaded Golgi-targeting platelet microparticle-mimetic nanoplatform (ATRA-Gol-PMMNPs)**	Retrograde transport	[232]
	**Doxorubicin (DOX)+retinoic acid (RA)-chondroitin-modified lipid nanoparticles (CSNs)**	High affinity compound surface modification	[233]

NMs can be encapsulated in proteinaceous carriers using specific proteins, such as lysosomal membrane proteins. These carriers can interact with the lysosomal membrane, facilitating the guidance of NMs into lysosomes. Ma and colleagues engineered AuNPs with different coatings, such as human gamma globulin (HGG), human serum albumin (HSA), or human serum fibrinogen (HSF). When these NPs were co-incubated with HeLa cells, they observed the adsorption of a protein layer on the surface of these AuNPs, forming a protein corona. The protein corona influenced the integrity and stability of the lysosomal membrane, leading to increased permeability [149].

Since lysosomes are the most acidic part of the cell, they can be targeted for delivery by using NPs that have acid-cleavable chemical bonds (such acetal or hydrazine) or by altering them, so they have pH-activatable characteristics. Zhong et al. demonstrated, for instance, that a PEG-PTTMA-PAA triblock copolymer may release doxorubicin hydrochloride (DOX·HCl) in response to changes in pH. Micelles are formed when PTTMA binds to PEG-PAA covalently via acetal linkages and the resulting triblock structure. DOX and PAA form electrostatic contacts, which allow DOX·HCl to be loaded into the micelles. Hydrolysis of the acetal bonds occurs when NPs enter lysosomes, causing micelles to disintegrate and drug release to occur [150]. To load rubyrin, Yu et al. created acid-activatable selenium NPs that target folate [151]. With the aid of folate ligands, the NMs are able to perform RME, which allows them to selectively infiltrate cancer cells and lysosomes. Penicillin G (PenG) prodrug nanocarriers created by Nicolas et al. exhibit remarkable intracellular antibacterial efficacy and are pH-sensitive. They linked squalene and PenG together via a cleavable connection that only forms at certain pH levels. The resultant PenG derivative self-assembled into nanocarriers due to its amphiphilic characteristics. Lysosomes got the final destination for these nanocarriers after they entered into cells via separate cytoplasmic and endocytic processes. Upon entering lysosomes, the NPs may be able to release PenG by rapidly hydrolyzing acid-reactive bonds inside the lysosomal context [152]. In addition, NPs drug delivery systems that incorporate multistage controlled release and target lysosomes have been created by scientists. As an example, a peptide micelle that is both pH-sensitive and charge-reversible was created by Han et al. for use in cancer chemotherapy drug administration. This micelle depends on a slow reaction to the somewhat acidic pH of the endo/lysosomal context and the external tumor microenvironment [153].

### 4.2 Cytoplasm

Cytoplasm possesses multiple functions vital for maintaining basic cellular activities, synthesizing biochemical substances, and storing materials. It is the region within the cell that includes organelles and the fluid matrix, playing a crucial role in supporting and regulating key cellular processes such as metabolism, protein synthesis, and degradation reactions. NMs can penetrate the CM and enter the cytoplasm through various pathways. These pathways can be broadly categorized into two classes, with one involving endocytic routes leading to endosomes. For instance, Shukla and colleagues developed a multi-component small interfering RNA (siRNA) nanocomplex for delivering siRNA to hepatic stellate cells (HSCs). Once internalized, the multi-component siRNA nanocomplex is enveloped in early endosomes. After escaping from the endosome, the cargo dissociates from the nanocomplex, remaining in the cytoplasm [154].

**Figure 3.**
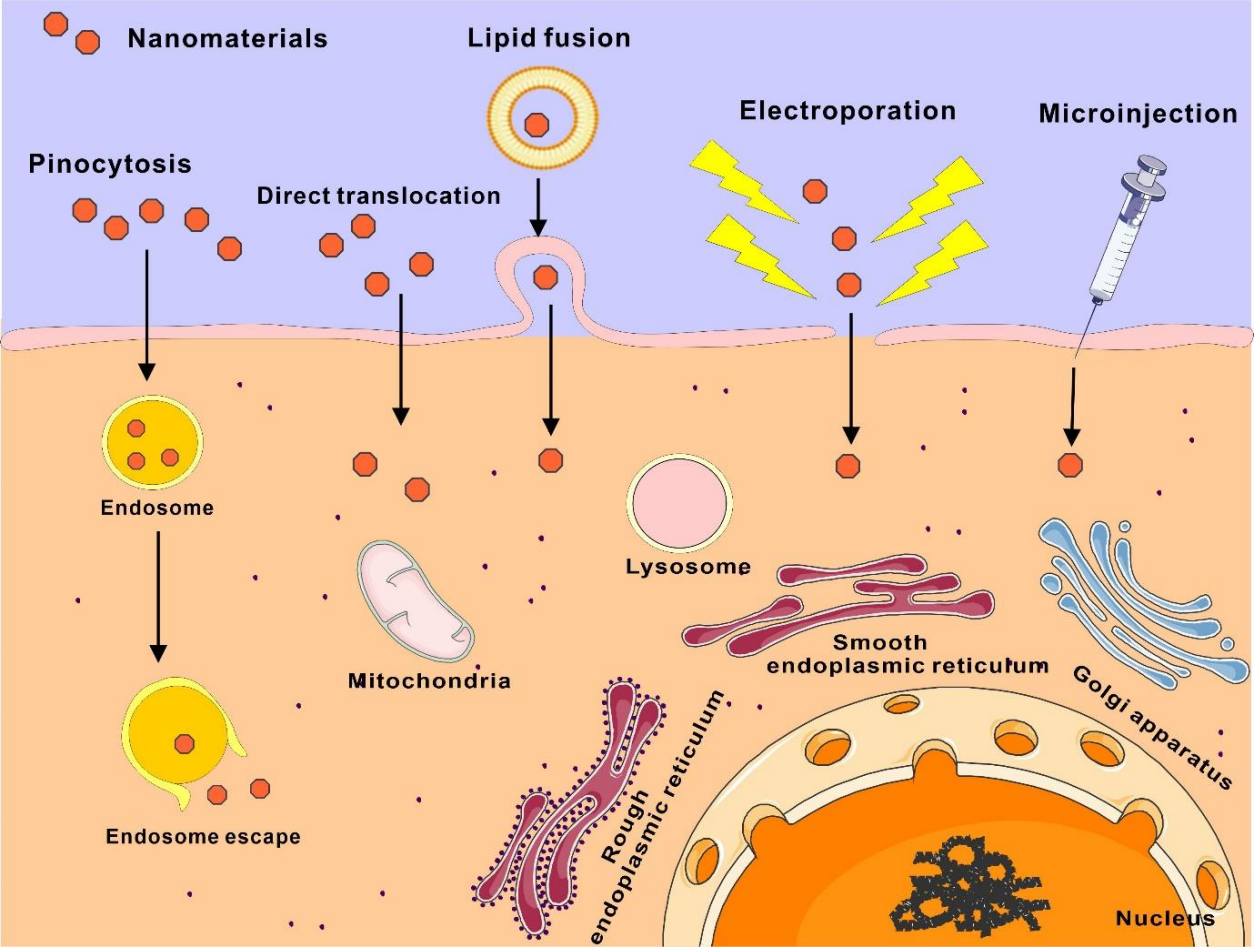
Approaches for nanomaterials to pass through the cell membrane to reach the cytoplasm.

Another approach involves passing through the CM directly into the cytoplasm via biochemical or physical pathways. This includes direct translocation pathways, lipid fusion, electroporation strategies, and microinjection strategies (Fig. 3). NMs can disrupt the CM by attaching to the lipid bilayer and entering the cytoplasm through direct translocation mechanisms. This pathway circumvents endosomal encapsulation and energy-dependent transport processes, enabling a direct entry into the interior of the cell [155]. Translocation was directly detected in red blood cells using semiconductor quantum dots measuring roughly 8 nm in size and possessing a zwitterionic surface [156]. The process of internalization did not lead to the creation of discernible pores in the cytoplasmic membrane. Another study found that zwitterionic AuNPs, which had a diameter of 2-4 nm, were able to directly diffuse across the plasma membrane of HeLa cells in vitro [157]. Lipid fusion is the phenomenon in which specific lipid bilayers merge with the CM [158]. After the fusing of the membrane, the substances contained within the NPs, such as proteins, nucleic acids, and small molecule payloads, are directly transported into the cytoplasm [159, 160]. Scientists have successfully suppressed genes by introducing siRNA into the cytoplasm via this specific mechanism [161]. Silicon NPs enclosed in fusogenic liposomes enable the fusing of lipids. Recent research indicates that NPs with amphiphilic organic shells and gold display lipid fusion behavior that is dependent on their size [162]. Electroporation is a process where electrical pulses are used to physically damage the CM, causing temporary pores to form. NPs can be transferred through these holes from the extracellular space to the cytoplasm. By carefully adjusting the electrical pulses, the creation of membrane pores through electroporation can be regulated to prevent any negative impact on cell survival [163]. NPs have been effectively utilized in electroporation procedures for imaging and genetic engineering purposes. Kim et al. employed mesoporous silica nanoparticles (MSNs)-coated hollow manganese oxide NPs for the purpose of labeling and monitoring adipose-derived mesenchymal stem cells. After applying an electric current of approximately 100 volts, the NPs successfully penetrated the mesenchymal stem cells. This was confirmed using MRI experiments conducted both in laboratory settings (in vitro) and in living organisms (in vivo) [164]. Another research conducted demonstrated the successful delivery of siRNA using lipid NPs by electroporation. This delivery method significantly suppressed the expression of PD-L1 and PD-L2 on human monocyte-derived dendritic cells [165]. Microinjection is the direct injection of small amounts of NPs into the cytoplasm using specialized microinjectors [166]. This approach effectively circumvents obstacles present in cells and the cellular membrane, enabling the injected NPs to rapidly penetrate the cytoplasm. Nevertheless, because NPs must be injected into each individual cell, the capacity of this method is restricted.

### 4.3 Nucleus

The cell nucleus is the central control center within a cell, primarily responsible for maintaining genetic stability and regulating gene expression and protein synthesis. Within the cell nucleus, chromatin is present, containing DNA molecules, and the nuclear envelope allows for the exchange of RNA and other molecules between the nucleus and cytoplasm through nuclear pores. Serving as a barrier to foreign entities entering the cell nucleus, the nuclear membrane separates the nucleus from the cytoplasm. Nuclear pore complexes (NPCs) dynamically move on the double membrane of the nuclear envelope. With a diameter of approximately 9-12 nm, nuclear pores are centrally located in each complex, regulating the flow of substances between the cytoplasm and the cell nucleus [167]. Nuclear pores provide a passive entry pathway for particles smaller than 9 nm, making them a potential target for molecules [168]. However, larger cargoes must enter the nucleus through active transport [169].

Tiny-sized NMs can enter the cell nucleus through passive nuclear transport, which depends on concentration gradients and does not require energy-driven processes. The NMs must be tiny enough to fit through the nuclear pores via passive diffusion. Huo and colleagues detected NPs ranging in size from 2-6 nm inside the cell nucleus, whereas NPs larger than 10-16 nm were located outside the nuclear compartment [168]. AuNPs with a diameter of roughly 4 nm are capable of penetrating the cell nucleus of breast cancer cells. However, AuNPs with an average diameter of around 14 nm are unable to pass through the nuclear membrane and are instead distributed throughout the cytoplasm [170]. For example, the cisplatin with a positive charge can go from the cytoplasm to the cell nucleus. In the nucleus, it can interact with the negatively charged DNA by electrostatic forces [171]. The size of NPs is a determining factor in their ability to enter the nucleus. Additionally, the shape of the NPs plays a key role in influencing the uptake of nanocomposites by the nucleus. Gaus and his colleagues conducted a thorough investigation of the cellular and subcellular behavior of NPs with various geometric shapes but identical surface properties. These NPs were composed of a block copolymer called poly (oligoethylene glycol methacrylate)-block-poly(styrene-co-p-vinylbenzaldehyde) [172]. The four shapes of NPs studied were micelles (20 nm × 20 nm), vesicles (100 nm × 100 nm), rods (5-10 × 100-300 nm), and worms (5-10 nm × 400-700 nm). The study found that the concentration of rod-shaped and worm-shaped particles in the nucleus was significantly higher than that of other particles compared to micelles and vesicles. However, only a limited number of NPs enter the cell nucleus through passive transport pathways.

**Figure 4.**
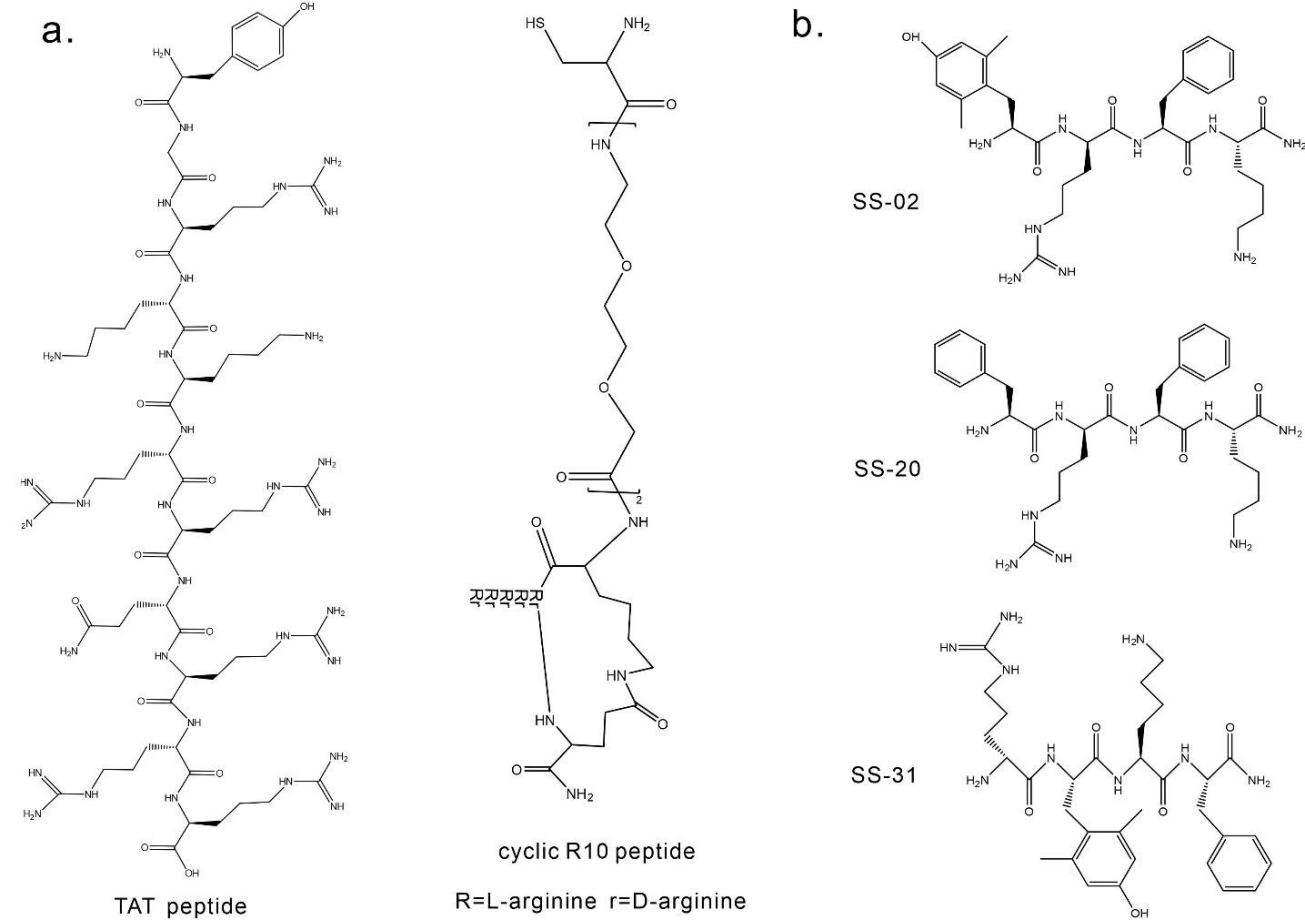
**Chemical structures of TAT peptide, cyclic R10 peptide, and SS peptides**. a) Chemical structures of TAT peptide and cyclic R10 peptide. b) Chemical structures of SS-02, SS-20, and SS-31.

To increase the proportion of NMs entering the cell nucleus, researchers have surface-modified NMs with nuclear-targeting compounds, enabling NMs to enter the cell nucleus through active transport [173]. Three of the most common nuclear-targeting compounds are cell-penetrating peptides (CPPs) (such as TAT peptide and cyclic R10 peptide, Fig. 4a), nuclear localization signals (NLS), and DNA aptamers. TAT, a high cationic peptide with the sequence YGRKKRRQRRR, was the first cell-penetrating peptide discovered and is produced by human immunodeficiency virus type 1 [174, 175]. The TAT peptide's positive charge and its capacity to penetrate the CM enable it to engage in electrostatic interactions with the ECM present on the cell surface. The ECM comprises negatively charged glycosaminoglycans, which have the potential to initiate endocytosis. After being internalized, the peptide can directly enter the cytoplasm and then proceed to the cell nucleus, bypassing the endosomal/lysosomal pathway [168]. Following fusing with the TAT peptide, NPs measuring an average diameter of 50 nm have been shown to actively travel across the NPCs [176]. The TAT peptide has the ability to actively transport tiny particles of MSN into the cell nucleus [177]. A cyclic cell-penetrating peptide known as cyclic R10 peptide has been documented to have superior targeting of the cell nucleus compared to linear or cyclic TAT peptides [175]. The NLS amino acid sequence comprises a limited amount of positively charged residues, specifically lysine or arginine residues [178]. Both NLS and TAT utilize the same technique to get access to the cell nucleus. However, NLS is advantageous as it does not cause unintended penetration of the CM, resulting in minimal side effects [179, 180]. Currently, the majority of reported NPs designed to reach the cell nucleus have a diameter that is less than 50 nm. The optimization of NLS enables the transportation of considerably larger nuclear proteins into the cell nucleus. As an example, Tamam's research group chose chitosan NPs with a size of 150 nm and changed their surface with eight-peptide NLS (CPKKKRKV) at various densities. This was done to investigate how well they can penetrate the nucleus [181]. The researchers discovered that NPs with a moderate density of NLS of 0.9 NLS per square nanometer, which is roughly equivalent to 16,000 NLS per particle, showed the greatest amount of accumulation in the nucleus. This accumulation was 3.7 times higher compared to NPs with a higher NLS density of 2 NLS per square nanometer. Excessive NLS density may cause spatial interference and competitive binding with importin proteins, resulting in decreased nuclear accumulation. To achieve the best effectiveness in the penetration of therapeutic drugs into the nuclear membrane of NPs, it is crucial to thoroughly optimize the density of NLS. Non-optimal binding orientations may lead to ineffective movement of the drugs into the nucleus. NMs have the ability to form strong associations with DNA aptamers that possess nuclear targeting properties. The DNA aptamers possess the ability to identify particular antigens located within the cell nucleus and then gain access to the nucleus with the assistance of antibodies [182]. Dam et al. utilized Au nanostars (AuNS) that were modified with aptamers specific to nuclear proteins (Apt-AuNS) [183]. Nucleolin is a protein that is phosphorylated in the nucleolus and is normally found in the nucleus of healthy cells. However, it is found in higher levels in the cytoplasm of cancer cells and moves to the CM. Hence, the pivotal importance lies in the transport of anticancer ligands from the cell surface to the cell nucleus. Dam et al. employed a 26-mer, 7.8 kDa single-stranded DNA aptamer called AS1411, together with AuNS that were treated with a chemotherapeutic medication. AS1411 disrupts many nuclear protein activities, inhibits DNA repair in the cell nucleus, destabilizes Bcl-2 mRNA, and triggers tumor cell death. Thanks to the nuclear proteins' shuttling characteristics, Apt-AuNS is efficiently carried into the cell nucleus, since these proteins facilitate the movement of these nanostructures to the perinuclear area of the cell.

### 4.4 Mitochondria

Mitochondria is a crucial cellular organelle responsible for energy production and are involved in various key cellular processes, such as regulating apoptosis, maintaining redox balance, and controlling intracellular calcium homeostasis [184]. Generally, some NMs can enter mitochondria, but this typically requires specific design and modification, as mitochondria have a unique double-membrane structure and highly selective permeability. The mitochondrial membrane is relatively impermeable to most substances, allowing only specific molecules to pass through. However, researchers have developed NMs and techniques that enable targeted entry into mitochondria to achieve specific therapeutic goals or intervene in mitochondrial functions. This includes using mitochondrial-targeting signal peptides, introducing specific chemical groups on the surface of NMs, and packaging NMs in mitochondria-specific lipid NPs, among other approaches.

Recent studies have indicated that there are three types of mitochondrial targeting signal peptides, including mitochondrial penetrating peptides (MPPs), Szeto-Schiller (SS) peptides (a class of cell-penetrating short peptides with antioxidant activity), and mitochondrial targeting sequence (MTS) [185]. MPPs are synthetic peptides (typically 4-16 amino acids in length) carrying cationic charges and hydrophobic amino acid residues. Their entry into mitochondria is driven by an electrochemical gradient, providing them with the ability to transport across CM and selectively localize in the mitochondrial matrix [186]. A range of anticancer drug-MPP peptide conjugates has been reported, for example, MPP linked to DOX through a succinic acid linkage [187, 188]. SS peptides consist of a short amino acid sequence composed of four alternating aromatic and basic amino acid residues (Tyr, Dmt, Phe, Arg, and Lys). Various SS peptides (SS-01 ~ SS-31) have been synthesized using these amino acids. Among them, SS-02 (Dmt-D-Arg-Phe-Lys- NH_2_), SS20 (Phe-D-Arg-Phe-Lys-NH_2_) and SS-31 (D-Arg-Lys-Phe-NH_2_) exhibit antioxidant properties due to the presence of aromatic amino acids such as Dmt and Tyr (Fig. 4b). Unreacted tyrosine or dimethyltyrosine radicals can react with superoxide radicals to form tyrosine hydroperoxide, thereby eliminating ROS [189, 190]. SS-31, along with other SS peptides, has progressed to clinical trials for patients with ischemia-reperfusion and microvascular injury [191]. MTS is a naturally occurring peptide that facilitates the transportation of proteins produced in the cytoplasm to the mitochondria. It does so by interacting with the mitochondrial import machinery located on both the exterior and interior mitochondrial membranes [192, 193]. Yamada et al. created a nanocarrier using liposomes that can specifically connect with MTS. This allows for the precise delivery of specific proteins to the mitochondria [194]. While MTS sequences possess the capacity to specifically target mitochondria, it has been documented that their ability to enter the CM is restricted. To address this problem, new research has proposed the integration of MTS with CPPs [195]. Li et al. developed a drug delivery system called P-DR8MTS, which specifically targets mitochondria. They achieved this by combining a mitochondrial targeting peptide (ALD5MTS) with a cell-penetrating peptide (octa-arginine or R8), and then assembling the peptide with the anticancer medication DOX [196]. The study found that the combination of mitochondrial-targeting sequences with CPPs effectively targets the mitochondria.

By introducing specific chemical moieties on the surface of NMs and leveraging the highly hydrophobic nature and strong negative membrane potential of the mitochondrial inner membrane, they can be guided into the mitochondria through electrostatic attraction. For instance, triphenylphosphine (TPP) derivatives are widely studied mitochondrial-targeting moieties, utilizing their strong lipophilicity and delocalized cationic properties to target the inner mitochondrial membrane. Delocalized cations play a crucial role in this process, believed to reduce the change in free energy associated with the transition of the mitochondrial inner membrane potential from the aqueous environment to the hydrophobic environment. Qu and his colleagues conjugated TPP to the surface of MSNs loaded with DOX [197]. The results indicate that the combination of cationic charge on the surface ligands and the binding of three lipophilic phenyl groups enables NPs to penetrate the mitochondrial membrane. Liu designed a conjugate system consisting of MLP, TPP, and Arg-Gly-Asp (RGD). This conjugate system, mediated by pH-responsive histidine, self-assembles into mitochondria-targeted NPs, enhancing tumor selectivity and antitumor efficacy through lysosomal escape [198]. Additionally, Jeena et al. synthesized a novel mitochondria-targeting peptide [199]. This mitochondria-targeting peptide, composed of β-sheet structures, forms aggregates in HeLa cells, binding with TPP and accumulating primarily in the mitochondria. The F16 cation is likewise a delocalized species that tends to concentrate within the mitochondrial matrix. F16 was identified in a high-throughput screening of a chemical library for chemicals that inhibit the growth of freshly formed breast epithelial cells [200]. The presence of F16 in mitochondria causes mitochondrial depolarization, disturbance of mitochondrial structure, and the opening of the mitochondrial permeability transition pore. Huan He et al. designed a novel dual-functional mitochondria-targeting anticancer agent (FPB) with both imaging capabilities and anticancer activity. FPB is formed by coupling the delocalized lipophilic cation F16 with the widely used fluorescence dye boron-dipyrromethene (BODIPY) using a benzyl linker. It selectively accumulates in the mitochondria of cancer cells, inducing apoptosis and causing cell death. Due to higher absorption by cancer cells, FPB exhibits increased toxicity towards cancer cells. FPB induces concentration-dependent elevation of superoxide levels, mitochondrial depolarization, and apoptosis in cancer cells, making it a dual-functional anticancer agent with selective anticancer activity and efficient imaging capabilities [201]. Rhodamine derivatives, such as Rhodamine 123 and Rhodamine 19, are also mitochondria-targeting agents because of their binding affinity to the mitochondrial membrane, subsequently disrupting the electron transport chain [202]. In src-transformed cells, Rhodamine 123 preferentially stains the mitochondria [203]. Mechanistically, its mitochondria targeting and accumulation are attributed to its lipophilicity and cationic nature, aiding its passage through the double mitochondrial membranes and retention within the negatively charged mitochondrial matrix. Similar to Rhodamine 123, Rhodamine 19 is also a potential mitochondria-targeting agent [204]. Rhodamine 19, through chemical modification, forms Rhodamine 19-drug conjugates, effectively replacing TPP and confirming its mitochondrial delivery capability [205]. Particularly, Rhodamine 19 possesses proton uncoupling activity, balancing the mitochondrial membrane, and is thus considered a mitochondria-targeting cationic uncoupler. Similarly, dequalinium (DQA), a single-chain amphiphilic molecule with two delocalized cationic centers, exhibits mitochondria-targeting characteristics. DQA, a lipophilic drug composed of two cationic quinoline groups, can be localized to the mitochondria of cancer cells [206]. The compound demonstrates anti-proliferative activity in vitro against various cancer cell lines and anti-tumor effects in vivo [207, 208]. Additionally, some other groups also exhibit mitochondria-targeting properties. Zhou et al. demonstrated that SWCNTs non-covalently functionalized with phospholipid-PEG could penetrate mitochondria by exploiting the mitochondrial membrane potential [209-211]. Furthermore, Ma et al. found that carboxylated SWCNTs could localize to mitochondria and regulate mitochondrial function [212]. In Vero cells, Hoshino used a 30-peptide extracted from cytochrome C oxidase to deliver quantum dots to mitochondria [213]. In another research, Ruoslahti utilized a dual amphiphilic pro-apoptotic _D_[KLAKLAK]_2_ peptide designed to destabilize mitochondrial membranes to target IONPs to the mitochondria [214].

Encapsulating NMs in mitochondria-specific lipid NPs, such as using liposomes or phospholipid NPs, enables interaction with the mitochondrial membrane, guiding the NMs to the mitochondria. Recent research has indicated that an innovative type of liposome, called MITO-Porter, is made up of 1,2-dioleoyl-sn-glycero-3-phosphoethanolamine (DOPE), sphingomyelin, or phosphatidic acid. This liposome has been modified on its surface with octaarginine (R8) and a pH-sensitive membrane fusion peptide known as GALA. This system facilitates the entry of chemicals into mitochondria via a process of membrane fusion. The system's high density facilitates its efficient internalization by cells by micropinocytosis and subsequent binding to mitochondria. In addition, GALA allows the carrier to exit from endosomes and enter the cytoplasm [215].

### 4.5 Endoplasmic Reticulum

ER is a eukaryotic cell organelle characterized by a network of membrane structures maintained by the cell's cytoskeleton. The ER is the primary organelle responsible for the proper folding of proteins. Partially folded proteins are retained in the ER, where, with the assistance of molecular chaperones and folding enzymes within the ER lumen, the folding process is completed. Alternatively, these proteins may be transported to the Golgi apparatus (GA) or lysosomes [216]. Due to the ER primarily consisting of a double-layered structure composed of phospholipids, cholesterol, and proteins, lipophilic molecules with specific affinity and charge can easily enter the ER. Xia et al. encapsulated saquinavir with a phospholipid bilayer to obtain lipid NPs [217]. Compared to simple drugs, the cellular uptake of lipid NPs is significantly increased, and the colocalization coefficient of lipid NPs in the ER reaches 0.83 at 1 h. This ER-targeted accumulation may be attributed to the interaction between the phospholipid bilayer coating and the ER membrane. Studies have suggested that, based on the nature of the ER, the goal of entering the ER can be achieved through specially designed and functionalized NMs. Wu et al. synthesized NPs (referred to as MNOCNPs, M = Ni, Pd, or Cu) through a hydrothermal reaction. The results showed that NPs modified with PEG exhibited high stability and good ER accumulation ability [218]. For instance, Shi et al. assembled amphiphilic quinoline ketone derivatives-peptide conjugates, namely quino-1-fmoc-racr, on nanodots, and the prepared nanodots were found to effectively enter the ER [219]. Mukai et al. synthesized poly (g-glutamic acid) NPs and, through confocal laser scanning microscopy analysis of cellular behavior, observed that poly (g-glutamic acid) accumulated in the ER lumen within 2 h [220].

Additionally, the connection of NPs with ER-targeting peptides or ER-retention signal sequences, such as the KDEL peptide [221] and ER-resident calcium-binding protein [222], also facilitates the targeted entry of NMs into the ER. The KDEL tetrapeptide sequence (Lys-Asp-Glu-Leu-COOH) targets the ER, and Acharya et al. developed AuNPs bound with the KDEL peptide, successfully delivering siRNA to the ER to inhibit the expression of nicotinamide adenine dinucleotide phosphate oxidase 4 [223]. Delie et al. prepared KDEL peptide-bound, paclitaxel-loaded functionalized NPs, finding enhanced ER targeting and anticancer effects [224]. Hadas et al. employed a peptide (KKXX signal) with a specific ER-targeting portion, previously demonstrated to target intracellular proteins to the ER [225, 226], to construct ER-targeting poly (DL-lactide-co-glycolide) (PLGA) NPs [227]. Confocal results demonstrated high colocalization of the NPs with the ER, indicating successful targeting. Moreover, compared to NPs without bound ER-targeting peptides, the cellular uptake of targeted NPs was significantly higher. Pardaxin (FAL) is a cationic antimicrobial peptide, derived from the red sea flat fish (Pardachirus marmoratus), which consists of a single polypeptide chain composed of 33 amino acids. Jian You et al. coupled the FAL peptide with gold nanospheres loaded with indocyanine green, designing the FAL-ichauins nanosystem, which demonstrated successful ER targeting in CT26 and B16 tumor mouse models [228].

### 4.6 Golgi apparatus

The GA is an organelle composed of numerous flattened vesicles. Its main functions include processing, sorting, packaging, and transporting proteins synthesized by the ER, then delivering them to specific locations within the cell or secreting them outside the cell. The trans-Golgi network (TGN) is the site where cargo is sorted before being directed to various cellular organelles or secreted outside the cell. There are two pathways for entry into the GA: anterograde transport and retrograde transport. NMs typically enter the GA through retrograde transport. For example, NMs can be covalently bound to the SLTB subunit [229]. Surface modification with compounds such as benzene sulfonamide, cysteine, chondroitin sulfate (CS), etc., which have a high affinity for the GA, can achieve the purpose of targeting the ER. Li et al. developed a prodrug nanoparticle targeting the GA by synthesizing retinoic acid (RA)- CS (CS-RA) [230]. This nanoparticle can accumulate in the GA of tumor cells and liver cells, releasing RA in acidic conditions. Luo et al. reported CS-modified nanomicelles loaded with DOX and RA, which can effectively target the GA of liver cells and release both DOX and RA. The research results demonstrate that these nanomicelles can effectively inhibit the activation of HSCs, thus treating liver fibrosis [231]. The GA has specific protein targeting signals that can be used to guide proteins to the GA. Researchers can incorporate these signal peptides onto the surface of NMs to achieve targeted delivery to the GA. Deng et al. used GA-targeted platelet-mimicking nanoplates (referred to as goll-pmmnps) as a nanoplatform, consisting of a nanoparticle core and a platelet membrane coating labeled with GA-targeting peptides. The goll-pmmnps targeted synovial fibroblasts in rheumatoid arthritis patients through integrin α2β1-mediated endocytosis and accumulated in the GA through retrograde transport [232]. NMs can be packaged into lipid NPs with GA-targeting properties. In this way, NMs can enter the GA through lipid packaging. Luo et al. synthesized CS-modified lipid NPs loaded with DOX and RA, which successfully entered the GA within cells and released DOX and RA to exert their effects [233].

## 5. Intracellular Trafficking and Cellular Retention of Nanomaterials

Trafficking and retention of NMs within cells are areas of significant focus in biomedical research. They involve understanding how NMs are taken up, transported, and retained within cells. This is crucial not only for nanodrug delivery, drug release, and cell therapy but also presents new opportunities in fields such as diagnostics, vaccine delivery, and gene editing. This section will delve into these aspects, revealing the transport pathways of NMs inside cells and how they are ultimately degraded or excreted. Additionally, factors influencing their excretion will be discussed.

### 5.1 Intracellular Trafficking

**Figure 5.**
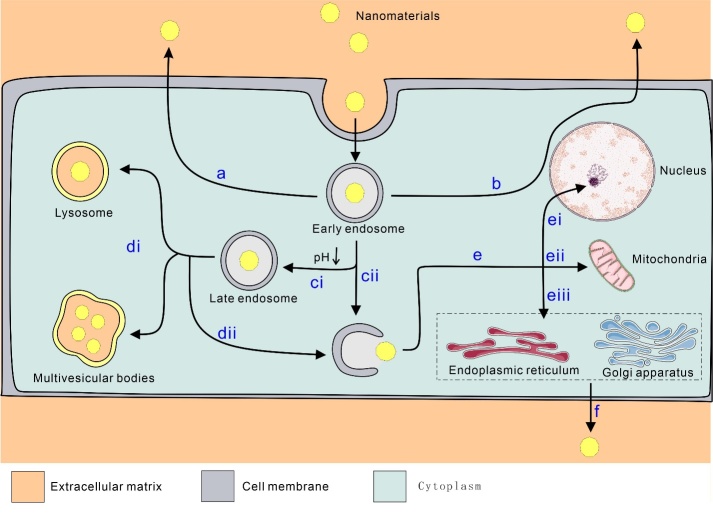
**The intracellular trafficking process of nanomaterials**. NMs typically first enter the early endosomes. a) Some NMs, along with endosomes, directly recycle to the CM. b) Some through the perinuclear region and recycle back to the CM. ci) Another portion may stay within early endosomes, slowly moving along microtubules towards the cell interior and fusing with the late endosomes. cii) A small portion of endosomes spontaneously degrade, releasing the enclosed NMs into the cytoplasm. di) Late endosomes may further fuse with lysosomes or multivesicular bodies, where NMs might undergo degradation under the acidic conditions of lysosomes. dii) Along the pathway to MVBs or lysosomes, some NMs may escape from vesicles into the cytoplasm. e) NMs located in the cytoplasm or within the cell nucleus may further enter the ei) nucleus, eii) mitochondria, eiii) ER and GA, possibly even undergoing fusion with the ER and GA. f) After entering the ER or GA, NMs can exit the cell through vesicles associated with the conventional secretory system.

Due to the complexity of the trafficking mechanisms of NMs within cells, describing all intracellular processes that occur after NMs uptake is challenging. The approximate process of intracellular NMs transport is depicted in the diagram (Fig. 5). Upon cellular uptake, NMs typically first enter the early endosomes, considered the primary sorting station for endocytosis. Some NMs, along with endosomes, recycle back to the CM through the perinuclear region and can also directly recycle to the CM. Enclosed NMs within endosomes may be subsequently expelled from the cell. Only a small portion of endosomes spontaneously degrade, releasing the enclosed NMs into the cytoplasm [12]. While another portion may stay within early endosomes, slowly moving along microtubules towards the cell interior and fusing with the late endosomes. Late endosomes may further fuse with lysosomes, where NMs might undergo degradation under the acidic conditions of lysosomes [100], but this is not necessarily the endpoint of intracellular transport. In some cases, cells may release undigested NMs through exocytosis [234]. Along the pathway to MVBs or lysosomes, some NMs may escape from vesicles into the cytoplasm, a process referred to as endosomal escape [235]. However, NMs located in the cytoplasm or within the cell nucleus may, through mechanisms not yet fully understood, further enter cellular organelles such as the nucleus, mitochondria, ER, and GA, possibly even undergoing fusion with the ER, GA, and other organelles [236]. After entering the ER or GA, NMs can exit the cell through vesicles associated with the conventional secretory system [237]. Meanwhile, NMs located in the cytoplasm can exit the cell either by re-entering the endosomal system or directly through non-specific mechanisms.

Typically, endosomal entrapment is a major barrier for NMs to exert their effects within cells. Once confined within endosomes, NMs may face degradation within lysosomes or direct expulsion, preventing them from realizing their intended biological effects and therapeutic functions. Therefore, endosomal escape is crucial for the intracellular delivery of NMs. Researchers have explored various methods to facilitate the effective escape of NMs from endosomes. Once NMs escape the endosomal compartments, they can interact with the cytoplasm and bind to various intracellular targets. The efficiency of endosomal escape can be enhanced by designing the physicochemical properties of NMs, such as surface charge and surface ligands. In one study, NMs modified with CPPs induced endosomal membrane rupture, allowing NMs to escape through the ruptured membranes [238]. While not fully comprehended, it is believed that the mechanism is triggered by the cationic surface modification of CPPs, resulting in membrane disruption through the proton sponge effect [239]. The proton sponge effect results from the reduction of late endosomal pH, causing an increase in internal proton concentration. Designed NMs with surface groups capable of absorbing protons trigger the entry of chloride ions into the endosome, causing an increase in endosomal osmotic pressure. Water then enters the endosome through osmosis, leading to endosomal rupture. Therefore, NMs can escape from the endosome. Dalal and colleagues encapsulated quantum dots in a polyacrylate shell, which was further modified with PEG and varying amounts of TAT peptides [240]. TAT peptide is a type of CPPs. According to Dalal et al., the distribution of NPs inside cells varies depending on the quantity of peptides attached to each nanoparticle. The fewer peptides attached to the nanoparticle surface, the more likely it is to enter the endosome and localize in the TNG and perinuclear region. On the contrary, the more peptides attached to the surface of NPs, the easier they are to extravasate into the cytoplasm. Similar results have been validated in Pan et al.'s research on peptide-conjugated MSNs for nucleus-targeted drug delivery [176]. This indicates the significance of membrane-destabilizing peptides in surface modification for nuclear escape. In addition to membrane-destabilizing peptides, scientists have also utilized other membrane-disruptive surface modifications. It has been reported that NPs modified with polymers such as PEI can induce endosomal rupture [241]. A study by the Irvine group reported an endosomal escape pathway. Initially, they fabricated 2-4 nm amphiphilic AuNPs embedded with a TGF-β inhibitor, followed by coating the NPs with antibodies targeting CD8+ T cells [242]. Transmission Electron Microscopy (TEM) analysis indicates that after 24 h of in vitro cultivation, these amphiphilic AuNPs traverse the endosomal membrane, effectively delivering the TGF-β inhibitor into the cytoplasm of CD8+ T cells. It can be concluded that the targeting antibodies are hydrolyzed in the endolysosomal system, and the amphiphilic AuNPs permeate the endosomal membrane through non-destructive membrane penetration, thereby delivering the TGF-β inhibitor into the cytoplasm to exert its function. A major characteristic of the endosome and lysosome within the cell is its acidic pH. Therefore, the acidic pH can be employed to facilitate the endosomal release of pH-sensitive NPs. Wang et al. linked DOX to 30 nm AuNPs via a hydrazone bond that is sensitive to low pH. This allowed them to transport DOX to cancer cells that have multidrug resistance [243]. Upon entry into cancer cells, the hydrazone link within the NPs is broken down as a result of the acidic pH conditions seen in the late endosome. This leads to the release of DOX from the core of the AuNPs into the cytoplasm.

### 5.2 Cellular Retention

Although the cellular uptake of NMs has been extensively studied with various applications and modifications, there is less definition regarding the cellular exocytosis and excretion of NMs. Similar to the cellular uptake of NMs, the cellular exocytosis and excretion of NMs also depend on the cell type, the physicochemical properties of the NPs, as well as the concentration of NMs and their incubation time with cells (Table 3).

The cellular retention and excretion of NMs seem to vary depending on the kind of cell. The aforementioned finding was derived from internalization experiments conducted on three distinct human cell lines: H1299 (human lung cancer), NE083 (human esophageal epithelial), and NL20 (human bronchial epithelial) utilizing 50 nm silica NPs [244]. Analysis of these three distinct cell lines demonstrated commonalities in the cellular absorption mechanism of NMs across the cell lines. However, the excretion profile of NPs is influenced by the cell line type. Researchers reported that the silica NPs stayed longer in esophageal epithelial cells compared to the time they stayed in lung cancer and bronchial epithelial cell lines. Furthermore, the release of MSNs from several mammalian cell lines demonstrated that the unique properties of different cell lines in retaining and eliminating MSNs could result in an uneven transmission of MSNs between cells [245]. Yanes et al. [246] examined how different cell types affect the rate of exocytosis of phosphate-modified MSNs (~130 nm). The researchers used MSNs extracted from various cell lines, including adenocarcinomic human alveolar basal epithelial cells (A549), breast cancer cell lines (MDA-MB231 and MCF-7), melanoma cell line (MDA-MB435), pancreatic cancer cell line (PANC-1), and human embryonic stem cells (H9). The rates of excretion of the NPs were 87%, 81%, 75%, 61%, 36%, and 4%, respectively. From these findings, it can be inferred that NPs with the same physicochemical characteristics demonstrate notable variations in their rates of cellular excretion across various cell types. Consequently, it is crucial to evaluate the cellular excretion or associated attributes of NMs for every distinct cell type.

**Table 3:** Impact of cellular retention of nanomaterials.

Nano-materials	Size	Cell type	Surface property	Time	Retention	Uptake	Exocytosis	Ref.
Silica nanoparticles	50 nm	H1299 HONE1 NL20	——	3 h	In endo-lysosomes or cytoplasm	——	——	[244]
				**10 h** **48 h**	In the endo-lysosomes			
**Mesoporous silica nanoparticles (MSNs)**	——	HUVECs HeLa	——	2 h	The amount of endocytosis reaches the platform	——	——	[245]
Phosphonate-modified nanoparticles (P-MSNs)	130 nm	A549	Phosphonat	2 h	——	——	87%	[246]
		**MDA-MB231**					81%	
		**PANC-1**					75%	
		**MCF-7**					61%	
		**MDA-MB 435**					36%	
		**H9**					4%	
**P-MSNs**	——	A549	Phosphonat	6 h	——	——	84%	
**Folate-MSNs**			Folate				66%	
**PEI-MSNs**			Polyethylen-imine (PEI)				49%	
Silica nanoparticles	60 nm	HepG2	——	——	Distribute on the cell membranes, in the cytoplasms and lysosomes	80.10%	63%	[248]
	**180 nm**					99.60%	67%	
	**370 nm**					97.60%	58%	
	**600 nm**					88.60%	38%	
**Poly (D, L-lactide-co-glycolide) nanoparticles**	97 ± 3 nm	VSMCs	——	——	High concentration (200 ug/ml) resulted in a 2.5-fold increased retention	——	——	[254]
Paclitaxel-Loaded Glycol Chitosan Self-Assembled Nanoparticles	300 nm	Hela	——	30 min	——	——	Many of the nanoparticles were exocytosed	[256]
				**1 h**				
				**6 h**			The amount of exocytosed nanoparticles decreased	

Research indicates that the size of NMs [247] can influence the exocytosis process and retention time of these particles within cells. Hu et al. [248] studied the exocytosis of MSNs of different sizes in liver cells (HepG2 cells). After several hours, the exocytosis rates for particles with sizes of 60, 180, 370, and 600 nm were 63%, 67%, 58%, and 38%, respectively. Jin et al. found that smaller SWCNTs in NIH-3T3 cells exhibited a faster exocytosis rate compared to larger SWCNTs [249]. These results illustrate the complex impact of NMs size on the extent of excretion and suggest that smaller NMs are more prone to release from cells.

The surface characteristics of NMs can influence the process of exocytosis and the duration of their retention within cells [250]. For example, Kolosnjaj-Tabi and colleagues monitored the progression of iron oxide-coated AuNPs in mice over a period of one year [251]. The NPs, which had a gold core with a diameter of 5 nm and were covered with iron oxide, exhibited an average diameter of 16 nm in a core-shell hybrid structure. The NPs underwent selective modification using either an amphiphilic polymer or a catechol-derived PEG ligand as surface ligands. Irrespective of the alteration of the surface, the NPs were internalized by cells. One year after being injected into the bloodstream, they were found in vesicles inside liver Kupffer cells and splenic Ito cells. As per the authors' findings, one year after administering NPs, the PEG-coated NPs with an iron oxide shell had undergone degradation and were cleared from the liver and spleen. On the other hand, the amphiphilic polymer-coated NPs still contained up to 10% of the initial iron oxide dose in these organs. In a separate investigation, Yanes et al. [246] examined the impact of various surface-modified MSNs - specifically phosphates, folic acid, and PEI-on the discharge of NPs from A549 cells. Following a duration of 6 h, the amounts of phosphate-MSNs, folic acid-MSNs, and PEI-MSNs released were 84%, 66%, and 49% correspondingly. These findings demonstrate that various surface changes significantly influence the exocytosis and excretion properties of NMs.

Another element affecting exocytosis and excretion is the concentration of NMs and the length of time they are incubated in cells. The uptake and elimination of NMs are active and coordinated processes, influenced by the concentration of NMs both within and outside the cells. According to reports, the rate of exocytosis is highest when the number of NMs inside the cells reaches its maximum or saturation level [252]. Specifically, the amount of silver NPs present in the liquid portion of human glioblastoma U251 cells is influenced by both the duration of incubation and the concentration of silver NPs. According to a publication, the amount of silver NPs released at a concentration of 400 μg/mL is three times greater than the amount released at 100 μg/mL [253]. Panyam et al. [254] examined how the duration of pre-incubation and the concentration of NMs affect the process of exocytosis of PLGA NPs in VSMCs. It was discovered that raising the concentration of NMs in the culture media from 100 μg/mL to 200 μg/mL led to a 2.5-fold rise in the percentage of NMs that were kept within the cells. In addition, they found no notable impact of pre-incubation duration on the excretion of NPs. Park and colleagues [255, 256] observed a decrease in the excretion of chitosan NPs from HeLa cells as the pre-incubation duration increased, ranging from 10 minutes to 6 h. The variations in cell types and NMs characteristics may account for these diverse outcomes.

To increase the therapeutic effects of NMs, researchers have been focusing on slowing the pace at which NMs are expelled from cells, hence prolonging their retention. Kim et al. utilized a supramolecular chemistry approach to prolong the exocytosis of AuNPs in MCF-7 cells [257]. MCF-7 cells took up AuNPs that were treated with quaternary amines. Later on, the technique of in situ treatment was employed to create complexes containing quaternary amine groups using cucurbit. This led to the clustering of AuNPs inside the cells. The densely clustered gold nanoparticle complexes remained isolated inside the TGN. TEM and inductively coupled plasma mass spectrometry investigations revealed that the aggregated particles were entirely prevented from being released outside the cell over a 24 h period, and there were no documented signs of harmful effects on cell viability. Efficiently isolating NMs from cells is of utmost importance, particularly in the development of nanomedicines, in order to mitigate any possible adverse reactions. Exocytosis is an essential element of the immunological process. immunological cells, including granulocytes, natural killer cells, and cytotoxic T cells, carry out immunological responses by releasing particles. More precisely, when cytotoxic lymphocytes are activated, they release particles that contain perforin. These particles create gaps in the membrane of the target cells, leading to the death of the cells [258]. Jones and his colleagues employed the exocytosis of cytotoxic lymphocytes to create NPs that respond to perforin [259]. The drug-loaded lipid NPs were affixed to the surface of cytotoxic T lymphocytes. When the antigen bound to the cytotoxic T lymphocytes, the secreted perforin broke down the nanoparticle shell, causing the drug to be released. Additional investigation into the exocytosis of NMs is necessary in order to facilitate the development of NMs by nanomedicine researchers with precise intracellular transport, pharmacokinetics, and exocytosis properties (Table 3).

## 6. Disease Applications and Implications

The application of NMs in the field of medicine holds great promise for diagnosing, treating, and understanding various diseases. Here we highlight a few important uses and consequences of NMs in the context of diseases, and Table 4 shows a summary of the applications of NMs in various diseases.

### 6. 1 Cancer

Compared with traditional diagnosis and treatment methods of cancer, NMs provide an alternative strategy for t tumor argeted therapy due to their unique physical and chemical properties, such as nanoscale size, large surface-to-volume ratio, adjustable surface characteristics, and the ability to encapsulate various drugs and control drug release [260]. For a long time, in the study of tumor targeted therapy, improving the efficiency of tumor targeting is the key issue and main technical difficulty in the development of high-quality tumor-targeted NMs [261]. Although existing studies have reported the wide application of various NMs in cancer therapy, there are still some limitations that need to be overcome, such as the interaction mechanism between NMs and cells, short blood circulation time, and poor aggregation in target tissues. The cellular internalization process of various NMs is a prerequisite for biological applications [262]. Therefore, understanding the uptake, localization and retention of NMs in cancer cells can provide a better reference for targeted cancer therapy.

The delivery of NMs to tumors is based on specific and non-specific cell interactions. Specific (active) targeting relies on the functionalization of the nanoparticle surface with ligands that complement the target, which may be peri-tumor and intracellular blood vessels, ECM, tumor cells, or intracellular targets. In non-specific (passive) targeting, there is a modifier on the surface of the NPs [263]. Mitochondria, nuclei, lysosomes, and ER are the main targets of NMs for cancer treatment, because their functions are closely related to cell growth, proliferation, differentiation, and death, and play a key role in regulating cell biology [264]. Studies have revealed that zinc oxide NPs enter human osteosarcoma cells through a VPS34/regulatory dynein 2-dependent endocytosis pathway, directly targeting and destroying mitochondria, and then being captured into lysosomes, thereby initiating mitophagy-mediated apoptosis [265]. The sericin-PBLG micelles designed by Guo et al can be effectively internalized into cells through CME. Sericin-pblg-adriamycin is transported to the perinuclear lysosome and accumulates in the perinuclear lysosome. DOX is then released in a low pH microenvironment. The released DOX directly enters the nucleus, causing DNA damage and enhanced anti-tumor effect [266]. In addition, Zhang and Pandey developed NMs that can effectively accumulate in ER regions through endocytosis mechanisms, leading to ER stress and subsequent apoptosis [267, 268]. To enhance the effects of permeability and retention (EPR) of cancer therapy, Liu et al reported a shape-changing nanomedical covered by macrophage CM I-P@NPs@M for the treatment of breast cancer. I-P@NPs@M retains its shapechanging ability after macrophage membrane coverage, promoting its retention in tumor areas and enhancing cell internalization, which has also been demonstrated at the cellular and animal levels. Biological distribution experiments have demonstrated that membrane camouflage prolongates circulation and provides a targeting effect for I-P@NPs@M, making it better for drug delivery to tumors [269]. The mesenchymal stromal cell-mediated photoactive NPs designed by Lenna et al can be internalized by mesenchymal cells within 1h, and the particles remain in the cells for at least 3 days. Results obtained in vitro and in vivo indicate that the NPs can inhibit the growth of osteosarcoma [270].

**Table 4:** The applications of nanomaterials in diseases.

Diseases	Cell type	Nanomaterials	Purpose	Endocytotic pathway	Fate/effect	Ref.
Cancer	Osteosarcoma cells	Zinc oxide nanoparticles (ZnO NPs)	As a potential nanomedicine for human osteosarcoma treatment	VPS34/dynamin 2-dependent endocytic pathway	In situ in mitochondria and destroyed mitochondria	[265]
	**MCF-7** **HepG2**	Polypeptide-based amphiphilic polymer containing hydrophilic sericin polypeptide backbone and PBLG side chains (Sericin-PBLG-DOX)	As a drug delivery	Clathrin-mediated endocytosis (CME)	Internalized into cells, accumulated in perinuclear lysosomes	[266]
	**BxPC-3** **HPSCs**	Cancer Cell Membrane-Camouflaged Nanorods	As a drug delivery	Caveolin-mediated pathway (CVME)	Accumulate in the endoplasmic reticulum (ER) region	[267]
	**HeLa**	Engineering Nanoparticles for Spatial Targeting of Endoplasmic Reticulum and Mitochondria (ER-Obt-NPs and Mito-Obt-NPs)	Targeting the endoplasmic reticulum and mitochondria to spatially disrupt the anti-apoptotic Bcl-2 protein in these organelles	These nanoparticles internalized specifically to mitochondria and ER through lysosomes	Selectively home into ER and mitochondria	[268]
Neurodegenerative Diseases	BV2	Europium doped cerium oxide nanoparticles (EuCeO_2_NPs)	As an immunomodulator for AD treatment	Phagocytosis, scavenger receptor upregulation	Located in endosomes and lysosomes	[274]
	**BV2** **SH-SY5Y** **NIH-3T3**	Cell membrane coated novel biomimetic CSPQ@CM nanoparticles	Targeting and modulating microglia to treat Parkinson’s disease (PD)	Through the specific interactions between the membrane surface vascular cells adhering to molecule-1 and α4β1 integrin expressed by microglia	Accumulation of nanoparticles in the substantia nigra and striatum of the brain	[275]
	**ATP13A2GBAXMEABE (2)-M17**	Poly (DL-lactide-co-glycolide) acidic nanoparticles (PLGA-aNP)	Restoring damaged lysosomal function and alleviating PD-related neurodegeneration in vivo	Pinocytosis, lysosomal-related vesicles	Taken up into cells and appro-priately targeted to lysosomes	[278]
**Infectious Diseases**	Human monocytes	A hydrophobic and lipophilic modified DTG prodrug is encapsulated into poloxamer nanoformulations (NMDTG)	To improve the human immunodeficiency virus type one (HIV-1) integrase strand transfer inhibitor dolutegravir (DTG) treatment outcomes	Macropinocytosis	Taken up by human monocyte-derived macrophages (MDM) residing for prolonged periods inside the cells	[282]
Cardiovascular Diseases	HL-1 Cardio-myocytes	Biocompatible and bioresorbable negatively charged calcium phosphate nanoparticles (CaP-NPs)	As a drug delivery	Conventional clathrin- and dyna-min-mediated endocytosis	Internalized into cardio-myocytes	[288]
	**H9C2** **A549, HUVECs** **RAW 264.7**	A reactive oxygen species (ROS)-scavenging material, antioxidative and anti-inflammatory nanoparticles (TPCD NP)	For targeted treatment of heart diseases.	The transcellular and/or paracellular pathways	Accumulate in the heart of mice by transport across the lung epithelial and endothelial barriers	[291]
	**H9C2**	PUE was loaded into mitochondria-targeted micelles (PUE@TPP/PEG-PE)	As a drug delivery	Triphenyl-phosphonium (TPP) cation enhance the cellular uptake, leading to absorptive endocytosis	Accumulate and retain in the ischemic myocardium.	[292]
Auto-immune Diseases	RAW 264.7 Primary intraperitoneal macrophages	Lipidoid-polymer hybrid nanoparticle (FS14-NP)	As a drug delivery	Natural phagocytosis of activated macrophages	Accumulate in macrophages within the arthritic joints	[295]
	**Raw 264.7**	Loaded MTX into nanoparticles of human serum albumin modified with mannose (MTX-M-NPs)	As a drug delivery	Receptor-mediated endocytosis (RME)	Accumulated mainly in arthritic joints	[296]
Metabolic Disorders	Caco-2	Adipose homing peptide (AHP)-functionalised GNPs (AHP-GNPs)	As a drug delivery	Receptor mediated endocytosis (RME)	Accumulate in the white adipose tissue	[300]
	**SVF**	Dibenzazepine-Loaded Nanoparticles (DBZ-NPs)	As a drug delivery	Internalized by both preadipocytes and mature adipocytes through the endocytotic pathway	Accumulate in the white adipose tissue	[301]
Respiratory Diseases	M2 macrophages	Exosome membrane of M2 macrophages and PLGA nanoparticles (PLGA NPs)	As a drug delivery	Macropinocytosis	Distributed in various organs, including the lungs, and targets M2 macrophages	[304]
	**BAL macrophages**	Gold nanoparticles (AuNP)	Nanocarrier delivery	Macropinocytosis	Attached to the epithelial surface, as well as within alveolar type I and type II epithelial cells	[308]
**Liver Diseases**	——	Nanoparticle (NP) with 14kd polycaprolactone (PCL) entrapping hepatitis B surface antigen (HBsAg) stabilized with Pluronics® F127PCL NPs	As oral delivery vehicle	Macropinocytosis	Accumulates in macrophages of small intestinal villi, peripheral lymph nodes and other reticuloendothelial organs	[315]
**Renal Diseases**	HK-2	Kidney-targeted rhein (RH)-loaded liponanoparticles (KLPPR) with a yolk-shell structure composed by polycaprolactone-polyethyleneimine (PCL-PEI)-based cores and kidney targeting peptide (KTP)-modified lipid layers	For renal diseases treatment using liponanoparticle delivery system	The non-lysosomal pathway of membrane fusion	Retained in the kidneys	[317]

### 6.2 Neurodegenerative Diseases

Neurodegenerative diseases are severe conditions affecting the neurological system, characterized by the progressive degeneration and impairment of nerve cells, ultimately resulting in the decline of nerve function. Glial cells play a dual function in the neurological system. Glial cells regulate and optimize the functional environment of neurons to safeguard them from the detrimental impacts of external influences. Nevertheless, excessive activation of glial cells upon stimulation can modify their initial protective impact, resulting in heightened harm [271]. Current studies suggest that NMs can be implicated in pathological alterations and disease development of the central nervous system through their actions on glial cells [272, 273]. During the initial phases of illness, some neuroprotective molecules may save neurons by diminishing inflammation in glial cells and eliminating harmful compounds present outside the cells. The team led by Machhi developed NMs of cerium oxide doped with europium. It was discovered that this NMs has the ability to enhance the phagocytosis capacity and Aβ breakdown capacity of microglia, while simultaneously decreasing the inflammatory response of microglia BV2 [274]. Liu et al. demonstrated the utilization of CM coated Cu_2_-xSe-PVP-Qe NPs for precise targeting and modulation of microglia in the treatment of Parkinson's disease (PD). The nanoparticle exhibits several enzyme activities, enabling efficient elimination of ROS, inducing the transformation of microglia into an anti-inflammatory M2-like phenotype, and mitigating neuroinflammation [275]. As the disease advances, aberrant glial cells secrete pro-inflammatory cytokines, chemokines, and intercellular adhesion molecules. These substances can worsen oxidative stress and inflammation, leading to more harm to neurons. Presently, existing research has documented that impairment of lysosomes results in lysosomal storage disorder and plays a role in the development of neurodegenerative disorders, including PD [276, 277]. Therefore, the approach of augmenting or repairing lysosomes seems to be a more effective treatment for the condition. Bourdenx et al. discovered that within 24 h, PLGA acid nanoparticles (PLGA-aNP) were delivered to lysosomes, limiting the alkalization of lysosomes following MPP+ treatment and reducing the harmful effects of MPP+ on cells. Through the process of repeatedly acidifying defective lysosomes after treatment with PLGA-aNP, lysosome function can be restored in various pathological conditions. Furthermore, the presence of PLGA-aNP can be observed in neurons in the brain following injection, and it effectively mitigates neurodegeneration associated with PD by repairing damaged lysosomes [278]. Furthermore, Zeng et al. documented analogous findings: Biodegradable poly (lactic-co-glycolic acid) nanoparticles (PLGA-aNP) measuring around 100 nm in diameter successfully targeted lysosomes. The NPs undergoes degradation and releases its acidic constituents within the lysosome. This process leads to acidification of the nearby lysosomal environment, thereby safeguarding neuronal PC12 cells against mitochondrial toxicity generated by MPP+ [279].

### 6.3 Infectious Diseases

Infectious diseases are a major driver of morbidity and mortality worldwide. Treatment of HIV, tuberculosis, and the Corona Virus Disease 2019 (COVID-19) epidemic of recent years is particularly challenging, as shown by the persistent spread and high mortality rates of these diseases. The use of NMs for antimicrobial preparation and the formulation of vaccines is expected to overcome the challenges associated with treating these diseases. In the treatment of HIV, long-acting injectable antiretroviral drugs are the most advanced nanotechnology in the clinical treatment of HIV. Long-acting injectable NPs reduce the frequency of dosing, thereby improving patient compliance, and they also allow medication to be administered to patients with dysphagia, resulting in viral suppression and improved patient life expectancy [280, 281]. For example, long-acting nanoformulations of dolutegravir (DTG), which encapsulate hydrophobic and lipophilic modified DTG prodrugs into poloxamer nanoformulations (NMDTG). NMDTG retained in the cytoplasm can release drugs from macrophages and inhibit viral replication and spread to CD4 + T cells [282]. In the treatment of tuberculosis, vaccination that promotes host immunity is the most effective intervention in the treatment of tuberculosis [283], and the use of NMs can better improve the immunogenicity of vaccines. For example, Tian et al. used DDA-MPLA-TDB liposomes to enhance the immunogenicity and protective efficacy of DNA vaccine against mycobacterium tuberculosis infection [284]. For the design and development of SARS-CoV-2 vaccine, basic research on nanobiotic interaction can be used to understand how SARS-CoV-2 infects cells and promote the development and application of new vaccines [285]. For example, SARS-CoV-2 is 60-140 nm in size, and SARS-CoV-2 infection relies on recognition and binding of the cell receptor human angiotensin-converting enzyme 2 (hACE2) through the receptor binding domain (RBD) of the spike protein. In the research and development of SARS-CoV-2 vaccine, the invasion of SARS-CoV-2 can be effectively inhibited by means of RBD inactivation and preventing RBD from binding with hACE2 [286].

### 6.4 Cardiovascular Diseases

Cardiovascular disease is the largest cause of death in the world, and the current drug treatment of cardiovascular disease still has considerable shortcomings, namely, low drug efficacy, side effects and drug tolerance dose [287]. One possible way to overcome the challenge of cardiovascular disease is to apply advanced nanotechnology to medicine and apply it to the field of targeted drug delivery to treat cardiovascular disease. Nanomedicine for cardiomyocytes is mainly focused on the improvement of myocardial infarction. After the NMs reach the myocardium, they must be internalized into the cardiomyocytes and escape from the kernal body or lysosome to avoid the degradation of the NMs and their loads. In order to enhance the pace at which cardiomyocytes are taken up by the body, the team led by Di Mauro V created biocompatible and bioabsorbable calcium phosphate NPs with a negative surface charge. These NPs are able to be successfully taken up by cardiomyocytes without causing additional harm or disrupting any of their normal functions. Calcium phosphate NPs were used to encapsulate synthetic microRNAs, which were then effectively transported to heart cells in both in vitro and in vivo experiments [288]. The research conducted by Savi demonstrated that cardiomyocytes may efficiently internalize negatively charged titanium dioxide NPs with a size of 50 nm [289]. Ferreira developed biodegradable porous Silicon NPs that are functionalized with atrial natriuretic peptide [290]. Targeting cardiac endocardial injury in particular, these NPs can enhance medication administration while preferentially targeting organs. It is possible for the NPs to bind to certain receptors on heart cells. Evidence demonstrates that it enhances the accumulation of drugs in the injured heart when given through the veins, and it also delivers the drug to the heart muscle after being administered throughout the body, resulting in biological consequences. The functionalized NPs gather and predominantly stay in the endocardial region of the injured heart, where they specifically control myocardial MAPK signaling. This offers novel perspectives on the advancement of specific treatments for cardiovascular conditions. Research has indicated that inhalable NPs enter and remove excessive ROS within heart muscle cells, known as cardiomyocytes, in order to reduce oxidative stress and cellular harm caused by DOX. Mice can deposit inhaled NPs in their hearts by passing through the pulmonary epithelial and endothelial barriers. Hence, the inhalation of NPs successfully suppressed DOX-induced cardiac failure in animals. NPs shown a notable improvement in their ability to specifically target the heart, efficiently enter cells, and localize within mitochondria. As a result, this boosted their therapeutic effectiveness [291]. In addition, puerarin has been loaded into mitochondria-targeting micelles to precisely deliver puerarin into mitochondria. In vitro results showed significantly enhanced uptake of H9c2 intracellular nanomicelles, which deliver puerarin to mitochondria and reduce lysosomal capture. The nano micelles have strong protective effect on H9c2 cell apoptosis induced by isoproterenol. In vivo tests have shown that nanomicelles can accumulate and retain in ischemic myocardenum [292].

### 6.5 Autoimmune Diseases

When it comes to autoimmune illnesses, NMs have a special place in treatment. The nanodrug delivery system has unique properties that allow it to extend the duration of action and decrease the frequency of administration. These include tiny particle size, large specific surface area, and strong adsorption [293]. Researchers in the field of rheumatoid arthritis have recently focused on creating targeted therapeutic medicines that, when paired with NMs, can effectively treat the disease [294]. To create polymer-lipid mixture NPs (FS14-NP), for instance, Song et al. injected a combination of spermidine lipids (S14) and F127 into an acetate buffer. NMs reduce ankle swelling, bone erosion, and cartilage destruction by passing through the "ELVIS" (Extravasation through leaky vasculature and subsequent inflammatory cell-mediated sequestration) effect, which is the selective accumulation of NMs in diseased joints through the natural phagocytosis of activated macrophages and delivering siIL-1β [295]. In order to direct methotrexate treatment to neutrophils, Lyu et al. developed a method to load the medication into mannose-modified HSA NPs (MTX-M-NPs). When compared to the identical NPs lacking mannose, neutrophil uptake of MTX-M-NPs was substantially better. Arthritic joints are the primary sites of MTX-M-NP accumulation. The retention of NPs in arthritic joints is enhanced by mannose-derived coatings. MTX-M-NPs is an effective RA medication delivery strategy since it decreases serum inflammatory cytokine levels, inflammation of the joints, and bone degradation [296].

### 6.6 Metabolic Disorders

The main mechanism by which NMs can be used to treat diabetes is that NMs can target pancreatic cells and activate glucokinase, thereby enhancing pancreatic insulin secretion and liver glucose uptake. Alkaladi and colleagues confirmed that zinc oxide and silver NPs can significantly reduce blood sugar in diabetic rats. Studies have shown that zinc oxide and silver NPs enhance the activity of serum insulin and glucokinase, and increase the expression levels of insulin, insulin receptor, glucose transporter 2 (GLUT-2) and glucokinase genes [297]. NMs can also be used as carriers to carry anti-diabetic compounds. Lipid NPs deliver siRNA of glucagon receptors in a mouse model of diabetes and show better results than leptin in reducing blood sugar levels in diabetes [298]. Chitosan-whey protein NPs containing tamarind trypsin inhibitors reduce fasting blood glucose and improve biochemical parameters associated with carbohydrate metabolism disorders without causing insulinemia [299]. NMs can promote the Browning of white fat cells, reduce the lipid content of fat cells, regulate the proliferation of fat cells, and thus treat obesity. For example, surface-modified AuNPs can effectively target fat cells and promote fat cell apoptosis. AuNPs coated with fat homing peptides can evade uptake by the reticuloendothelial system and show binding specificity for white adipose tissue (WAT) [300]. Jiang et al. developed the Dibenzazepine-Preadipocytes and mature adipocytes efficiently absorb loaded NPs through the endocytic route and suppress Notch signaling in adipocytes. Crucially, when these NPs were injected directly into the WAT groin area of mice that were made obese by their diet, the NPs stayed in that area and caused the fat cells to undergo browning. This led to better regulation of glucose levels and a decrease in body weight in the treated mice [301].

### 6.7 Respiratory Diseases

Asthma is a respiratory condition marked by temporary blockage of the airways, increased sensitivity of the bronchial tubes, and ongoing inflammation of the airways [302]. The anti-inflammatory effects of NPs have previously been shown in various inflammatory diseases. NPs increase therapeutic efficacy by facilitating drug delivery to target tissues, thereby improving drug deposition in the lungs [303]. Research has shown that PLGA binding NPs and the natural exosome membrane of M2 macrophages can alleviate inflammation in the lungs and ameliorate asthma in mice. After being injected intravenously, the NPs were dispersed throughout the body, including the lungs. There, they lingered for almost 48 h, specifically targeting M2 macrophages. While this was going on, NPs had no appreciable effect on host cell immunity [304]. In the study of Du et al., Montelukast sodium can improve the lung function of asthma patients, and has important roles in anti-inflammatory and immune function regulation. The use of GO nanocarriers to assist the deepening and enrichment of drugs at the site of lung inflammation. Effective mitochondrial targeted drug delivery is further achieved, thereby enhancing the inhibitory effect of anti-apoptotic proteins, leading to apoptosis of inflammatory cells [305]. Chronic obstructive pulmonary disease (COPD) is a chronic inflammatory lung disease that can cause airway obstruction [306]. The selection of NMs drugs for COPD can effectively target the diseased tissue microenvironment and have fewer side effects [307]. In the treatment of chronic obstructive pulmonary disease, inhalation nanocarriers for local or systemic treatment are promising. Geiser's study showed that inhaled AuNPs rapidly bound to alveolar epithelial cells in both Wt and Scnn1b-Tg mice. Compared with Wt mice, AuNPs uptake by surface macrophages of Scnn1b-Tg mice was lower, and the particle internalization rate of alveolar type I epithelial cells was higher. This may promote AuNPs deep translocations in Scnn1b-Tg mice, including enhanced epithelial targeting. These results suggest that AuNPs nanocarrier delivery is a successful strategy for the treatment of COPD alveolar epithelial cells and macrophages [308]. Mohamed et al. used polymer NPs that deliver mirnas to treat COPD and observed the internalization of NP in the A146 cell line. The results demonstrated the potential of polymer NPs as a delivery system for miR-146a for COPD [309].

### 6.8 Liver Diseases

The use of NMs as drug carriers in the treatment of liver diseases can protect drugs from degradation, promote the specific uptake of liver cell types, reduce the accumulation of drugs in tissues outside the liver, and overcome the drug resistance mechanism [310]. Activation of hematopoietic stem cells in chronically injured liver is responsible for the progression of cirrhosis. There have been studies using a variety of nano-agents to target and control the activation of hematopoietic stem cells in injured liver. For example, non-toxic inorganic NPs, such as cerium oxide, gold and silver, have been used to combat cirrhosis [311]. Wu et al. prepared galactosylated chitosan betulinate NPs, which showed liver targeting potential in cell uptake behavior studies and in vivo bioimaging tests. NPs can be absorbed and retained by HSCs, and can reduce the degree of liver injury in mouse models of liver fibrosis [312]. Studies have also reported that phosphatidylserine -modified curcumin NPs enhance the retention time of curcumin in serum and the ability to deliver curcumin in vivo to the liver. The levels of liver disease markers and pro-inflammatory cytokines in serum were reduced, thus effectively inhibiting liver fibrosis [313]. In the treatment of hepatitis, the use of NMs can effectively interact with immune cells to help restore immune cell function and reduce inflammation [314]. For example, Dinda et al. prepared polycaprolactone (PCL) hepatitis B surface antigen NPs and used them as oral delivery vectors for the therapy of hepatitis B. After oral administration, NPs with antigens are found in macrophages of intestinal villi, peripheral lymph nodes, and other reticuloendothelial organs with prolonged antibody responses, which might minimize the requirement for dosage boosters [315].

### 6.9 Renal Diseases

Because the drug delivery platform of NMs enables therapeutic agents to specifically accumulate at the diseased site, thereby reducing adverse systemic effects, some efforts have also been made to improve the treatment of kidney disease through targeted delivery of NMs. For example, Williams designed the renal tubule PLGA-PEG drug delivery system, which is administered intravenously, that can specifically target the kidney and remain in the renal epithelium for weeks. After being endocytosed by peritubular endothelial cells and then ingested by tubular epithelial cells, NPs can be released into the tubular interstitium [316]. Wang et al. designed kidney-targeted rhein-loaded liponanoparticles (KLPPR) with a yolk-shell structure composed of PCL-PEI -based cores and kidney targeting peptide -modified lipid layers. KLPPR ranges in size from 30 nm to 80 nm, and is distributed to the kidney through the glomerular filtration membrane. Kidney targeting peptide modification promotes uptake and endocytosis of renal cells through non-lysosomal pathways. Moreover, in vivo and in vitro experiments have verified that the nanoparticle has good biocompatibility, promotes the recovery of renal physiological function, and provides a promising strategy for the delivery system to treat kidney diseases [317].

## 7. Challenges and Future Directions

Nanomedicine, a burgeoning field at the intersection of nanoscience and medicine, holds immense promise for revolutionizing healthcare. However, its widespread application faces a myriad of challenges that necessitate careful consideration and thorough investigation.

In the field of nanomedicine, the contact and interaction between NMs and cells are key research focuses. The surface properties of NMs significantly influences cellular behavior. The surface characteristics of NMs can impact the orientation of cell growth in tissues, a phenomenon known as the "contact guidance" effect. With the advancement of nanotechnology, researchers can engineer specific structures to control a range of cellular behaviors, including adhesion, proliferation, migration, and differentiation, meeting the needs of tissue engineering and regenerative medicine. These techniques hold promising applications in the repair and regeneration of highly oriented tissues such as myocardium, neural tissues, and tendons [318]. However, the intricate interactions between NMs and biological systems at the cellular and molecular levels are not fully understood. This lack of comprehensive understanding can impede the development of effective and safe NMs. Therefore, understanding the interactions between NMs and biological systems is a key focus for future research. The biocompatibility of NMs is a critical factor in ensuring their acceptance within biological systems. Numerous studies indicate that the size, surface properties, and shape of NMs directly influence their biocompatibility. Materials in the size range of 10 nm to 200 nm can effectively avoid triggering immune clearance, demonstrating good potential for prolonged circulation in the body [319]. The shape of NMs can affect the activity of macrophages, as seen in LPS-induced macrophages, where only long needle-like carbon nanotubes can induce the expression of IL-1α, suggesting potential toxic risks [320]. By adding specific functional groups/surfactants/polymers to the surface of NMs, such as polyethylene glycol polymers, which are generally considered to have good biocompatibility, NMs can be widely used for modification, achieving targeted drug delivery with minimal adverse reactions [321]. However, the potential toxicity of NMs with long-term exposure and accumulation in biological systems remains a mystery. Future research directions will focus on designing NMs to maximize their biocompatibility, mitigate potential toxicity, and explore new methods for biocompatibility assessment. The stability and degradation rate of NMs in the body play a crucial role in drug delivery. Too rapid degradation may lead to premature drug release, while too slow degradation may hinder material excretion, increasing potential toxicity. Certain inorganic NMs tend to accumulate significantly in the reticuloendothelial system, posing significant toxicity issues and limiting clinical translation [322]. However, for nanomedicine carriers, it is advisable to avoid rapid in vivo degradation and premature drug release. For instance, Fe_3_O_4_ hollow NPs with a 3 nm pore size can achieve controlled and sustained release of cisplatin through pore diffusion, resulting in effective drug delivery and cancer treatment outcomes [323]. Therefore, future research should focus on designing NMs with appropriate degradation rates and stability to maintain ideal drug release kinetics in the body. The entry of NMs into the biological system may trigger immune responses and inflammation, affecting their drug delivery and biocompatibility. For example, bio-polymeric fillers, carbon-based NMs, and porous metals cannot achieve good biocompatibility due to their induction of innate immune reactions and persistent inflammatory responses, impacting the complete repair of damaged musculoskeletal tissues [324]. Hu et al. reported a novel drug delivery agent that combines vesicles derived from red blood cell membranes with PLGA polymer NPs, reducing the body's immune response and demonstrating better drug delivery efficacy than NPs coated with PEG [325]. Addressing this issue, future research should focus on a deeper understanding of the interaction between NMs and the immune system, as well as how to mitigate immune responses and inflammation to enhance the controllability and predictability of NMs in therapy.

The effectiveness of NMs in disease treatment depends on their targeting effects on specific cells or tissues. For diseases like cancer, the efficacy of drug delivery relies heavily on the biological distribution of the drug or drug carrier. The drug must reach specific biological targets with minimal systemic distribution. NMs have the ability to deliver drugs to target tissues, cells, or organs more specifically and effectively, enhancing treatment efficiency [326]. Therefore, the targeting effects of NMs on specific cells or tissues determine their effectiveness in disease treatment. There are reports suggesting that nano-lipid carriers loaded with ganoderic acid can interact with various cancer signal proteins, demonstrating better anti-tumor effects compared to the drug alone [327]. Additionally, studies by Kirpotin et al. indicate that targeted portions on the surface of anti-HER2 immunoliposomes significantly increase the capture rate of NPs in tumor cells expressing HER2 compared to non-targeted liposomes, achieving intracellular drug delivery [328]. However, the current targeting efficiency of NMs in vivo still faces challenges. Future research needs to focus on improving the specificity of NMs to ensure accurate and efficient delivery to target sites while minimizing the impact on normal tissues. New targeting strategies, such as surface modification and bio-labeling, will be crucial in achieving better targeting effects. Effective drug delivery requires precise control of drug release from NMs to maintain appropriate therapeutic concentrations. With the development of drug carriers of NMs, they can be designed to achieve controlled release, optimizing drug delivery for improved precision therapy [329]. There are reports indicating that the release curve of DOX can be adjusted by changing the opening size of silica nanocarriers loaded with DOX [330]. Li et al. synthesized a NMs with a surface cell membrane coating that can be controllably released through photocatalytic degradation, aiding in photodynamic therapy for killing tumor cells [331]. Currently, the release kinetics and dose control of nanomedicine remain complex issues. Future research should focus on developing controlled release systems that allow adjustment of drug concentrations during treatment and ensure sustained therapeutic effects, thereby reducing side effects. As NMs are widely applied, the issue of drug resistance is gradually becoming prominent. Future research needs to address the mechanisms of drug resistance that NMs induce during long-term treatment and seek solutions to mitigate this problem.

The large-scale preparation and manufacturing of NMs remain challenging. Future research needs to focus on the standardization and scaling of manufacturing methods to ensure the consistency and scalability of nanomedicines. New manufacturing technologies and processes will be crucial in achieving this goal. The application of NMs involves regulatory and ethical issues, including their potential impact on human health and the environment. Future studies should delve deeper into safety standards for NMs and formulate relevant regulatory policies. Additionally, ethical concerns, such as privacy and informed consent, also require increased attention. The release and emissions of NMs may potentially impact the environment. Future research should emphasize the behavior and effects of NMs in the environment to ensure their sustainability and environmental friendliness.

## 8. Conclusion

The research findings regarding the cellular uptake, subcellular localization, and cellular retention of NMs play a pivotal role in advancing nanoscience and its applications. Initially, the characteristics of NMs, such as surface properties, size, and shape, exert a profound influence on their cellular uptake mechanisms. Subsequently, NMs display highly specific subcellular localization, offering vast potential for precise therapy and bioimaging. Lastly, the regulation of intracellular retention holds paramount importance for nanomedicine delivery and long-term therapies, achievable through tailored modifications to the properties of NMs.

These pivotal discoveries underscore the necessity for thorough exploration of the interaction between NMs and cells, contributing to the refinement of methods and technologies in the realms of medicine and biomedicine. They not only present increased opportunities and optimism for the future of healthcare but also create avenues for the development of safer, more effective treatment methodologies, and diagnostic tools. The continual innovation and rapid progression of NMs will persistently influence the medical landscape, providing inventive solutions for the treatment and monitoring of diverse diseases. Consequently, we advocate for sustained in-depth research in this field to unlock the boundless potential of NMs in biomedicine, fostering further innovations in healthcare and the medical domain.
